# Experimental autoimmune encephalomyelitis pathogenesis alters along animal age: impact of S100B expression

**DOI:** 10.1007/s11481-025-10195-5

**Published:** 2025-04-14

**Authors:** Ana Rita Ribeiro, Raquel Pereira, Catarina Barros, Andreia Barateiro, Ainhoa Alberro, Afonso P. Basto, Luís Graça, Maria Vaz Pinto, Fábio M. F. Santos, Pedro M. P. Gois, Susan E. Howlett, Adelaide Fernandes

**Affiliations:** 1 Faculdade de Farmácia, Research Institute for Medicines (iMed.ULisboa), Universidade de Lisboa, Lisbon, Portugal; 2 Departamento de Ciências Farmacêuticas E Do Medicamento, Faculdade de Farmácia, Universidade de Lisboa, Lisbon, Portugal; 3 IIS Biogipuzkoa Health Research Institute, San Sebastian, Spain; 4 Gulbenkian Institute for Molecular Medicine, Lisbon, Portugal; 5 CIISA – Centro de Investigação Interdisciplinar em Sanidade Animal, Faculdade de Medicina Veterinária, Universidade de Lisboa, Lisbon, Portugal; 6 Laboratório Associado Para a Ciência Animal E Veterinária (AL4AnimalS), Lisbon, Portugal; 7 Faculdade de Medicina, Universidade de Lisboa, Lisbon, Portugal; 8 Department of Pharmacology, Dalhousie University, Halifax, NS Canada; 9 Department of Medicine (Geriatric Medicine), Dalhousie University, Halifax, NS Canada

**Keywords:** Age, Experimental autoimmune encephalomyelitis, Late-onset multiple sclerosis, Multiple sclerosis, S100B

## Abstract

Multiple Sclerosis (MS) is the leading inflammatory and non-traumatic cause of disability in young adults, with late-onset MS emerging in middle-aged patients often resulting in poorer treatment responses and worse prognoses. The calcium-binding protein S100B is elevated in MS patients, and its targeting has shown promise in reducing disease severity in experimental autoimmune encephalomyelitis (EAE) models. However, most studies on MS pathology have focused on young animal models, leaving a gap in understanding the effects of age and S100B ablation on disease progression throughout the lifespan. This study aimed to characterize EAE in mice of different ages, examining demyelination, inflammation, and immune responses to determine whether S100B ablation could mitigate MS pathogenesis across the lifespan. EAE was induced in six cohorts of C57BL/6 mice: young adults (3 months), older adults (6 months), and middle-aged (12 months), including corresponding S100B knockout (KO) groups, followed for 23 days. Upon sacrifice, spinal cords were assessed via immunohistochemistry and Real-Time qPCR, while splenocytes were analyzed for immune cell characterization. Results indicated a more severe disease course in 12-month-old mice, marked by increased gliosis, inflammation, and impaired microglial phagocytic activity. Notably, S100B absence reduced gliosis and inflammatory markers across all ages, with 12-month-old S100B KO mice showing increased regulatory T cells. These findings highlight the exacerbating role of age and elevated S100B in MS progression, underscoring the importance of identifying age-specific MS markers and therapeutic targets.

**Graphical Abstract:**

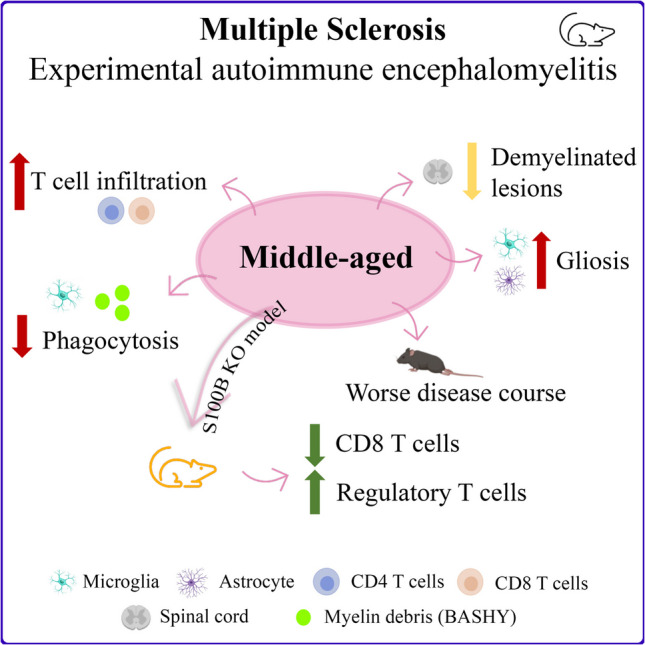

**Supplementary Information:**

The online version contains supplementary material available at 10.1007/s11481-025-10195-5.

## Introduction

Multiple Sclerosis (MS) is a chronic demyelinating and progressive autoimmune disease of the central nervous system (CNS), being the most common inflammatory and non-traumatic cause of disability in young adults (Dobson and Giovannoni 2019). The age of onset is extremely variable. Although in most cases MS is diagnosed between the ages of 20 and 49, it may also be diagnosed at older ages (above 50 years), being defined as late-onset MS, and accounting for 0.6 to 1.2% of the MS patients (Buscarinu et al. 2022; Capasso et al. 2023). Late onset MS has become particularly important due to the increase in life expectancy and the availability of effective disease modifying therapies (DMTs) in MS (Dema et al. 2021; Macaron et al. 2023). Most patients present with a relapsing–remitting MS form at early age of onset, whereas patients with late-onset develop progressive forms, which are linked to worse disease outcomes and poor response to treatments (Vaughn et al. 2019). Late-onset is also characterized by a higher prevalence of motor disability, sensory disturbance, visual impairments and immunosenescence (Vaughn et al. 2019; Capasso et al. 2023) accompanied by more spinal and fewer cerebellar lesions when compared to early-onset MS patients (Kis et al. 2008). Previous studies by our group used a novel frailty index (FI) tool in the in vivo experimental autoimmune encephalomyelitis (EAE) mouse model of MS to show that age significantly increases FI scores with more contribution from motor symptom parameters such as paralysis, impaired strength, and poor coordination (Ribeiro et al. 2022).

Pathological hallmarks of MS include the accumulation of demyelinating lesions in the spinal cord and brain due to the entrance of peripheral immune cells into the CNS. This phenomenon increases the production of inflammatory mediators and activates astrocytes and microglia (Filippi et al. 2018). Moreover, the release of pro-inflammatory cytokines increases permeabilization of the blood–brain barrier, attracting more immune cells that exacerbate CNS inflammation (Dema et al. 2021). The inflammatory environment and the loss of myelin sheaths promote oligodendrocyte and neuronal death, ultimately leading to neurodegeneration (Dendrou et al. 2015). In fact, although it is clear that immune system senescence increases with age (Papadopoulos et al. 2020), less is known about the impact of age on immune cell responses in MS and in disease progression.

S100B, a Ca^2+^ binding protein mainly produced and released by astrocytes in the CNS, has been described as a potential biomarker of MS disease pathology and also as a potential therapeutic target (Barateiro et al. 2016). Previous studies from our group have demonstrated that augmented S100B levels are present at the time of diagnosis in relapsing–remitting MS patients (Barateiro et al. 2016). Moreover, we also showed that S100B blockade by pentamidine protected against oligodendrogenesis impairment and neuronal loss by reducing the density of astrocytes and increasing microglia with regenerative properties (Barros et al. 2022). Interestingly, the same study showed that S100B ablation reduced EAE motor symptoms and associated glial reactivity, demyelination and inflammation in 11-week-old mice (Barros et al. 2022). The importance of S100B has been studied in a variety of neurodegenerative disorders (Michetti et al. 2019), but its role in aging is poorly understood. Indeed, in healthy aging, S100B levels are correlated to distinct brain regions and increased expression is found in the cerebrospinal fluid of older people (Portela et al. 2002). At advanced age, increased expression of S100B is associated with the production of proinflammatory cytokines with detrimental impacts on CNS tissues (Langeh and Singh 2020). However, the relationship between late-onset MS and the levels of S100B is unclear.

The objective of this study was to evaluate the impact of age in the in vivo EAE mouse model of MS with respect to glial reactivity, spinal cord gene expression profile and immune system response, and to correlate these events with the more severe disease phenotype as reported previously (Ribeiro et al. 2022). Moreover, we aimed to understand whether S100B ablation had a beneficial effect on EAE pathogenesis at different ages. In the present study, we demonstrated that age may be a predictor of worse disease outcomes, resulting in microglia dysfunction and adverse alterations in immune responses. We also showed that S100B depletion ameliorates the pathological hallmarks of MS in EAE mice at different ages in terms of glial reactivity, as well as inflammatory and regulatory immune responses.

## Materials and Methods

### Animals

Experiments used 3-, 6- and 12-month-old female C57BL/6 mice that were acquired from Charles River Laboratories and kept at the Gulbenkian Institute for Molecular Medicine (Lisbon, Portugal) animal facility. S100B knockout (KO) mice progenitors were purchased from the Jackson Laboratory. The colonies were established in Gulbenkian Institute for Molecular Medicine, and the genotyping was done by polymerase chain reaction (PCR), in which three pairs of primers were used to amplify different DNA regions according to the manufacturer’s instructions (Wellcome Trust Sanger Institute, Hinxton Cambridge, UK), as previously reported (Barros et al. 2022).

The animals were maintained in a controlled environment at 21ºC with 55–67% humidity, on 12-h light/dark cycle and were given food and water ad libitum. Before starting the experiments, mice were allowed to acclimatize for at least one week to adapt to the new environment and minimize animal stress. Animal care followed the recommendations of the European Convention for the Protection of Vertebrate Animals Used for Experimental and other Scientific Purposes (Council Directive 86/609/EEC) and National Law 1005/92 (rules for protection of experimental animals). All animal procedures were approved by the Institutional Animal Care and Use Committee and the National Animal Affairs Regulatory Office (Direção Geral de Alimentação e Veterinária). The best efforts were made to minimize the number of animals used and reduce their suffering.

### EAE Induction

C57BL/6 mice were divided into two groups: control and EAE. We induced EAE in 6 separate cohorts of mice: young adult (3-month-old), older adult (6-month-old) and middle-aged (12-month-old); young adult S100B KO (3-month-old), older adult S100B KO (6-month-old) and middle-aged S100B KO (12-month-old). Mice ages were defined according to Harrison and colleagues’ studies where they stablished how lifespan phases correspond to those in humans (Flurkey et al. 2007). Accordingly, the authors defined middle age in mice as ranging from 10 to 14 months, corresponding to 38–47 years of age in humans.

EAE was induced by active immunization following manufacturer’s instructions (Hooke Laboratories, Inc., #EK-2110). On day 0, EAE mice were immunized by subcutaneous injection of 125 µg (1.25 mg/mL) of myelin oligodendrocyte glycoprotein (MOG)_35–55_ peptide emulsified in complete Freund’s adjuvant (CFA) supplemented with 4 mg/ml of heat-inactivated *Mycobacterium tuberculosis*. The MOG_35–55_/CFA emulsion (100 µL) was injected in both the upper and lower back of each mouse. Additionally, the mice were injected 2 h and 24 h later with 100 µl of Pertussis Toxin (PTx; 200 ng/100 µL in phosphate buffer; intraperitoneal injection), to disrupt the blood–brain barrier. Control groups received the emulsion without the MOG_35–55_ peptide and were also injected with PTx.

On each of the 23 days following EAE-induction, mice were weighed and monitored for paralysis according to the standardized 5-point clinical scale as follows: limp tail – score 1; limp tail and weakness of the hindlimbs – score 2; limp tail and complete paralysis of the hindlimbs – score 3; limp tail, complete hindlimb paralysis and partial forelimb paralysis – score 4; complete paralysis – score 5 (Berard et al. 2010). Mice were also monitored for the recently published 34-item EAE—Clinical Frailty Index (FI) scale as previously described by us (includes physical condition, neuromusculoskeletal system, sensorimotor reflexes, paralysis/weakness, grip strength, ataxia/coordination, integument, self-care/grooming, vestibulocochlear/auditory system, ocular/nasal system, digestive system, urogenital system, respiratory system, discomfort, body temperature and body weight) (Ribeiro et al. 2022). Euthanasia was performed prior to the end of the experiment if a mouse scored 4 on two consecutive days or immediately if a mouse scored 5 according to the standardized 5-point clinical scale.

### Preparation of Splenocytes and Flow Cytometry

Animals were sacrificed at 23 days post induction (dpi) by anaesthetization with isoflurane and intracardially perfused with cold phosphate-buffered saline (PBS). Spleens were collected to isolate peripheral mononuclear cells. Briefly, tissue was dissociated to obtain a cell homogenate. The mononuclear and the red blood cells were collected by centrifugation. Then red blood cells were lysed by adding ammonium-chloride-potassium (ACK) lysis buffer and the mononuclear cells isolated upon centrifugation. Afterwards, mononuclear cells were resuspended in staining solution and counted. Staining with Live/Dead Fixable Aqua Dead (Invitrogen #L34957) was performed before fixation to allow gating of viable cells. To prevent non-specific antibody capture by the Fc receptors, cells were incubated with anti-CD16/32 (clone 2.4G2) prior to staining. Single-cell suspensions were blocked for 20 min and stained for antibodies targeting specific surface markers for different lymphocytes: CD3 (Brilliant Violet 711 anti-mouse, BioLegend #100,241), CD4 (monoclonal antibody (RM4-5), PerCP-Cyanine5.5, eBioscience, Invitrogen #45–0042–82), CD8 (monoclonal antibody (53–6.7), APC-eFluor 780, eBioscience, Invitrogen #47–0081–82), CD25 (monoclonal antibody (PC61.5), Alexa Fluor 488, eBioscience, Invitrogen #53–0251-82) and Foxp3 (monoclonal antibody (FJK-16 s), eFluor 450, eBioscience, Invitrogen #48–5773-82). Single stainings and fluorescence minus one (FMO) control tubes were used to compensate and set the gates for the analysis. The gating strategy and the representative plots are shown in Supplementary Fig. 1. Samples were acquired on a FACSCanto and analyzed with FCS Express™.

### Histological Analysis

To perform histological procedures, the lumbar region of the spinal cord and a brain hemisphere were fixed in 4% paraformaldehyde (PFA) at 4ºC. Next, samples were cryopreserved with 30% sucrose in PBS and then, snap-frozen in TissueTek® OCT™ compound (Sakura Finetek Europe) and kept at −80ºC until sectioning. Samples were cross-sectioned as serial transversal and sagittal cryostat sections for spinal cord and brain hemisphere respectively (20 μm thickness) at −20ºC (Cryostat Leica CM S3050) and collected on Superfrost Plus glass slides.

After lumbar spinal cord and brain cryosectioning, the slices collected on glass slides were stained for Luxol fast blue (LFB) and hematoxylin staining and visualized by light microscopy to assess demyelination and cellular infiltration, respectively. Briefly, slices were hydrated with PBS and incubated with chloroform and 100% ethanol misture (1:1) for four hours at room temperature (RT) and then rinsed in a 95% ethanol solution. After that, slices were incubated with 0.1% LFB solution in 96% ethanol and 10% glacial acetic acid, overnight at 56ºC. To clean excess stain, slices were quickly rinsed with 96% ethanol followed by distilled water. For tissue differentiation, slices were rinsed in a 0.1% lithium carbonate solution followed by 70% ethanol and distilled water. These steps were repeated until the white and gray matter (WM and GM, respectively) were distinguishable. For tissue counterstaining, slices were incubated with hematoxylin for six minutes, at RT. Then, slices were washed with running water for five minutes. Hydrochloric acid was used to differentiate the slices followed by tap water one last time, for five minutes. In the end, dried samples were mounted with Entellan (Merck, #K12572938). The images were taken with an optical microscope, Leica DC 100 camera (Leica) with 10 × magnification under a bright field in a total of 3 slices per animal. The extent of lesions was quantitatively evaluated by determining the percentage of spinal cord area that showed lower LFB staining (demyelination) and increased hematoxylin staining (cell infiltration), using ImageJ (Fiji Is Just) software.

### Immunohistochemistry and Data Analysis

After spinal cord sectioning, frozen sections were defrosted at RT and post-fixed with 4% PFA for 10 min followed by washing three times with PBS (10 min each). Then, slices were permeabilized with 0.25% Triton X-100 in PBS for 10 min and incubated with blocking solution containing 5% bovine serum albumin, 5% fetal bovine serum and 0.1% Triton X-100 in PBS for one hour, at RT. After blocking, slices were probed with the primary antibodies diluted in the blocking solution for approximately 48 h, at 4ºC: rat myelin basic protein (MBP, 1:200, BioRad, #MCA409S), rabbit ionized calcium-binding adapter molecule 1 (Iba1, 1:250, Wako, #019–19741), mouse glial fibrillary acidic protein (GFAP, 1:100, NovoCastra, #6,035,278), rabbit Neurofilament 200 (NF200, 1:200, Sigma, #4142), rabbit CD4 (1:100, abcam, #133,616), and mouse CD8 (1:100, Dako, #M710301-2). Following incubation, slices were washed three times for 10 min each with PBS and incubated with the appropriate secondary fluorescence antibodies diluted in the blocking solution (1:500) for approximately two hours, at RT: Alexa 488 anti-rabbit; Alexa 594 anti-rat; and Alexa 647 anti-mouse (all from ThermoFischer). Next, slices were washed three times for 10 min each with PBS and incubated with 4’,6-diamidino-2-phenylindole (DAPI) in PBS (1:1000, approximately five minutes) to stain cell nuclei. For myelin debris staining, the BASHY probe was used as described previously by our group (Pinto et al. 2021). Finally, slices were washed three times for five minutes each with PBS and mounted using Fluoromount-G (Southern Biotech) for fluorescence/confocal microscopy. Fluorescent images were obtained by confocal microscopy using Leica DMi8-CS inverted microscope with Leica LasX software under 20 × and 40 × magnification. Approximately 18–20 stacks were taken per slice, for a total of 3 slices per animal, and the analyses were performed on merged z-stacks. Demyelination and inflammation were analyzed in WM and GM. Moreover, the percentage of area occupied by Iba1 and GFAP was measured using ImageJ (Fiji is Just) software. To assess glial activation (Iba1 and GFAP) and cell infiltration in white matter lesions, demyelinated lesions were analyzed. Regions of interest corresponding to the demyelinated plaque (lesioned area, P), the adjacent periplaque (PP) and the normal appearing white matter (NAWM) were established by cell nuclei density and MBP immunostaining as follows: the plaque of demyelinated lesions was characterized by the high density of cell nuclei and no myelin staining; the PP was determined by the area corresponding to a perimeter of 100 μm measured from lesion edge (P) to the adjacent; finally, the NAWM was established as the area corresponding to a perimeter of 100 μm measured from the edge of the PP to the deep white matter. To assess axonal degeneration, NF200 positive cells were counted in demyelinating lesions and in the NAWM. To assess T cell infiltration, CD4 and CD8 positive cells were counted in demyelinating lesions. To assess myelin debris phagocytosis by microglia, BASHY and Iba1 areas were measured, as well as the colocalization of Iba1 with BASHY in lesions. Results are the average determined from three different slices from each of five mice per group.

### Semi-quantitative qReal-Time PCR

Total RNA was extracted from the thoracic spinal cord, from each experimental group, using RiboZol™ reagent, according to the manufacturer instructions (VWR Life Science). Total RNA was quantified using Nanodrop® ND-100 Spectrophotometer (NanoDrop Technologies) and reversibly transcribed into cDNA with the Xpert cDNA Synthesis Mastermix Kit (GRiSP), under recommended conditions. Quantitative Real-Time PCR (qReal-Time PCR) was performed using ribosomal protein (RP) L19 and RP L29 as endogenous controls to normalize the expression level of genes analyzed (Supplementary Table 1). qReal-Time PCR was performed on a QuantStudio™ 7 Flex Real-Time PCR System (Applied Biosystems), using a Xpert Fast SYBR MasterMix (GRISP, Research Solutions). qReal-Time PCR was performed in 384-well plates, with each sample performed in duplicate, and under optimized conditions: 50 °C for 2 min, 95 °C for 2 min, followed by 40 amplification cycles at 95 °C for 5 s and 62 °C for 30 s. To verify the specificity of the amplification, a melt-curve analysis was performed immediately after the amplification protocol (95ºC for 15 s, followed by 60ºC for 30 s and 95ºC for 15 s). Fold change was determined by the 2^−ΔΔCT^ method. After endogenous gene normalization, fold change for each experimental group were normalized to control 3-month-old mice followed by normalization of the EAE induced mice to the respective control group.

### Western Blot

To characterize protein expression, total protein was extracted with the RiboZol™ reagent, according to the manufacturer instructions (VWR Life Science). Protein pellets were resuspended in an 8 M urea (in 1 M Tris HCl, pH 8) and 1% SDS (1:1) followed by sonication and centrifugation at 3200 g for 10 min. Total protein concentration was measured using Nanodrop® ND-100 Spectrophotometer (NanoDrop Technologies) and stored at −80ºC. Protein samples were prepared with buffer containing 8 M urea (in 1 M Tris HCL, pH 8) and 1% SDS (1:1) followed by heating for 5 min, at 100ºC. The samples were loaded in equal amounts, separated on a Tris-Tricine gel and then, transferred to a nitrocellulose membrane (Amersham Biosciences, Piscataway, NJ, USA). The membranes were incubated in blocking solution with 1% Tween 20-Tris buffered saline (T-TBS) in 5% (w/v) non-fat milk, for one hour at RT. Then, the membranes were incubated overnight at 4ºC with the primary antibodies: rabbit complement component 1q (C1q, 1:500, abcam, #ab182451), goat complement component 3 (C3, 1:1000, Invitrogen, #PA1-29,715), rabbit S100B (1:250, DAKO, # Z0311) and mouse β-actin (1:10,000, Sigma, #A5441). On the following day, membranes were washed and incubated in the same blocking buffer with the secondary antibody (1:5000) for one hour, at RT: anti-rabbit HRP (Santa Cruz Biotechnology, #sc-2004), anti-goat HRP (Santa Cruz Biotechnology, #sc-2768) and ani-mouse HRP (Santa Cruz Biotechnology, #sc-2004). After washing, the membranes were incubated using Western Bright Sirius reagent (Advansta, Menlo Park) for one minute. The bands were detected and visualized in Invitrogen™ iBright™ CL 1500 equipment (Thermo Fisher Scientific) equipment. The relative intensities of the protein bands were analyzed using iBright analysis software (Thermo Fisher Scientific). Results were normalized to the expression of β-actin. The membranes were reused, following incubation with 0.2 M NaOH stripping solution for five minutes, at RT, in order to start a new immunoblotting protocol.

### Statistical Analysis

All results are presented as mean ± SEM. Data analysis was performed using PRISM GraphPad 8.0 (GraphPad Software). Significant differences between two groups were determined by unpaired two-tailed Student’s t-test; otherwise, a Mann Whitney U-test was used. To assess significant differences between more than two groups and between parameters, one-way and two-way ANOVA with a Tukey post-hoc test for multiple comparisons were performed. Differences between groups were considered statistically significant for *p < *0.05. Principal component analysis was carried out using PRISM GraphPad 9.0 (GraphPad Software).

## Results

### Middle-aged EAE Mice Show a Decreased White Matter Lesioned Area in the Spinal Cord

**Fig. 1:**
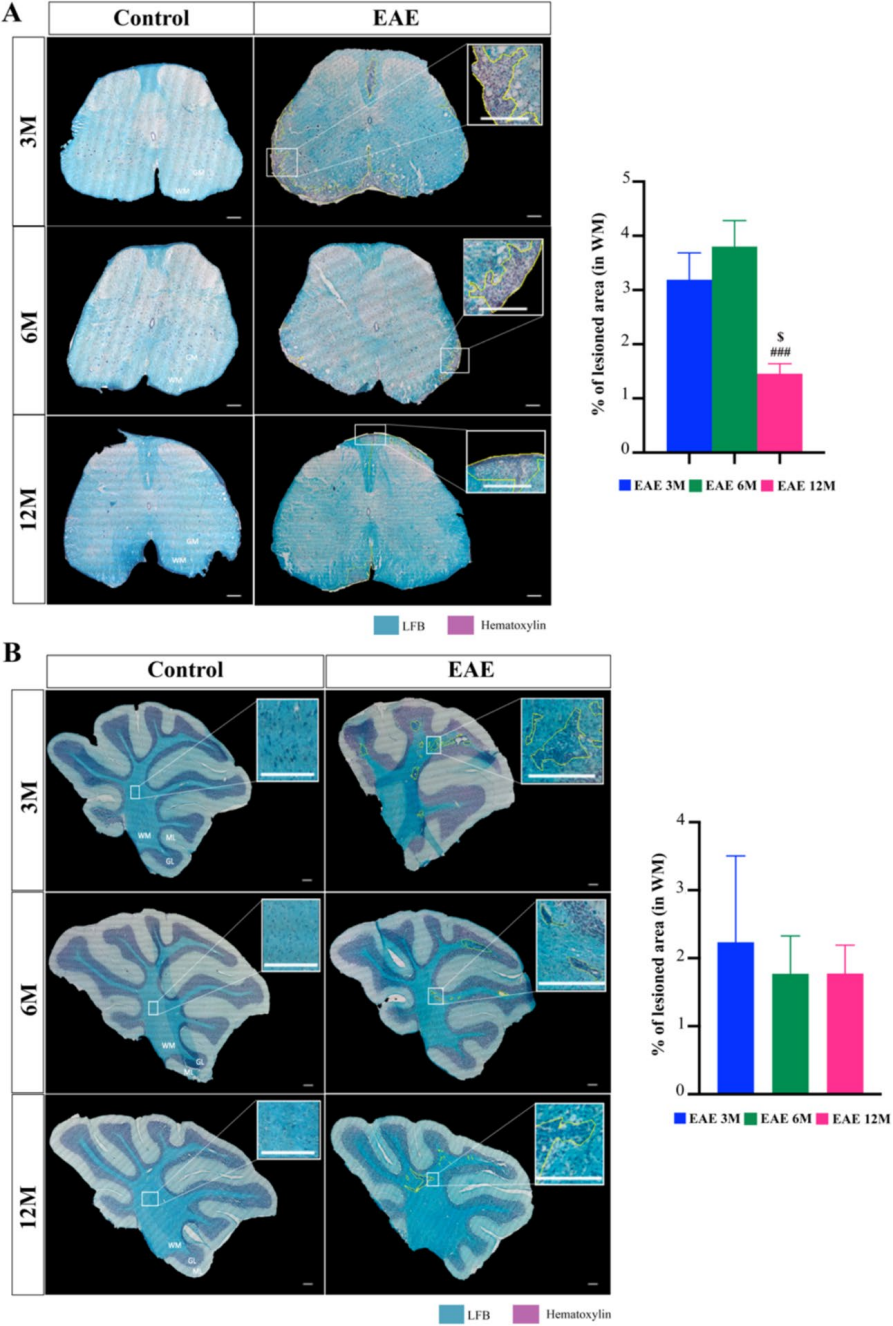
Experimental autoimmune encephalomyelitis (EAE) induction did not differentially affect the cerebellum lesion area of EAE mice at different ages, but decrease that of spinal cord in middle-aged mice. Female C57BL/6 mice at 3-, 6- and 12-month-old (M) were induced with EAE by MOG_35–55_ immunization and followed for 23 days post-EAE induction; lumbar spinal cords and cerebellum were collected at the end of the experiment. Representative images of Luxol fast blue (LFB) and hematoxylin staining of spinal cord (**A**) and cerebellum (**B**) in both control and EAE mice. Control mice have no lesions. Graph bars represent the percentage of lesioned area calculated in relation to total area of white matter (WM) in spinal cord and cerebellum assessed by LFB and counterstained with hematoxylin. Scale bar: 100 µm. Magnification: 10x. Results are expressed as mean ± SEM of one independent experiment, *n = *5 per group for each experiment and were analysed by Two-way ANOVA with multiple comparisons. GM – Grey matter; WM – White matter; GL – Granular layer; ML – Molecular layer. $ *p < *0.05 vs. 3-month-old EAE mice; ### *p < *0.001 vs. 6-month-old EAE mice

We previously showed that clinical symptoms of active immunization of EAE WT mice increase with age in 12-month-old mice showing atypical signs, including wobbling, early belly drag and splayed hindlegs, that may be detected by evaluation of the FI rather than the classical 5-point clinical score scale (Ribeiro et al. 2022). Recently Atkinson et al. demonstrated that middle-aged mice develop an accelerated and exacerbated clinical course of EAE when this model is induced by encephalitogenic CD4^+^ T cells (Atkinson et al. 2022). However, information on how the neuropathological features of the disease vary with age in the active immunization model was not explored. Here we explored the effects of age on the course of active immunization EAE and, given the different symptoms across ages, we evaluated both spinal cord and cerebellum pathology specifically for demyelinated lesions as shown in Fig. 1. Regarding demyelinated lesions, shown by the loss of LFB staining, age-matched control groups showed no lesions (Fig. 1A), as expected. However, 12-month-old EAE mice had a surprising and significantly lower percentage of lesioned area in spinal cord WM compared to 3-month-old and 6-month-old EAE mice (Fig. 1A, *p < *0.05 and *p < *0.001, respectively). Based on our previous findings showing an age-dependent phenotype (Ribeiro et al. 2022), we next hypothesized that EAE lesions would be increased in other CNS regions, namely in the cerebellum. However, we did not find any significant changes in the percentage of cerebellar lesions at the three different ages in EAE WT mice (Fig. 1B).

Overall, our results suggest that, although there is an age-distinct phenotype of EAE WT mice whereby older mice are more severely impaired, their atypical symptoms observed with the EAE-Clinical FI do not correlate with increased lesion areas or specific regional (e.g. spinal cord *vs.* cerebellum) susceptibility.

### Middle-aged EAE Mice have an Increased Glial Reactivity in Spinal Cord Demyelinated Lesions

Along demyelination, MS and EAE pathogenesis are characterized by activation of both astrocytes and microglia (Aharoni et al. 2021; Distéfano-Gagné et al. 2023). In pathological conditions, these cells become active leading to the exacerbated release of pro-inflammatory and neurotoxic factors that promote further demyelination and neurodegeneration. Indeed, both glial cells are highly present in demyelinated lesions and their evaluation is important to obtain a complete characterization of the EAE course. Therefore, we explored lesion glial reactivity in spinal cord and cerebellum.

**Fig. 2:**
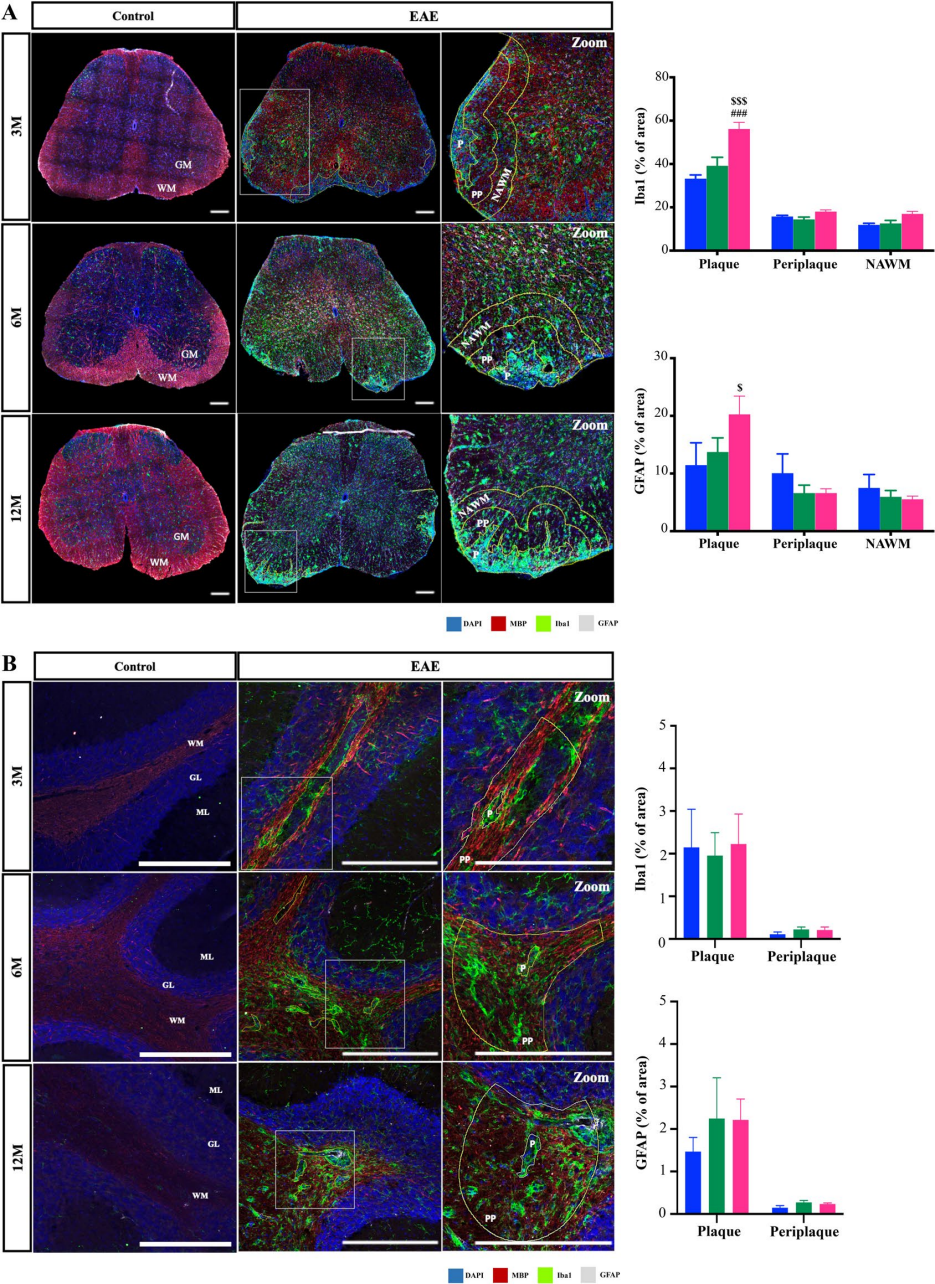
Middle-aged mice exhibit increased glial reactivity in the spinal cord. Female C57BL/6 mice at 3-, 6- and 12-month-old (M) were induced with experimental autoimmune encephalomyelitis (EAE) by MOG_35–55_ immunization and followed for 23 days post-EAE induction; lumbar spinal cords and cerebellum were collected at the end of the experiment and processed for immunohistochemistry. **A** Representative images of spinal cord sections showing lesioned area, the plaque (P), delineated by increased nuclei (DAPI—blue staining) without myelin (MBP—red staining). Periplaque (PP) and normal appearing white matter (NAWM) were delineated from the plaque area. Microglia were identified by Iba1 (green) staining and astrocytes by GFAP (white) staining. Graph bars represent the percentage of area stained for microglia and astrocytes. **B** Representative images of cerebellum sections showing P and PP. Microglia were identified by Iba1 (green) staining and astrocytes by GFAP (white) staining. Graph bars represent the percentage of area stained for microglia and astrocytes. Scale bar: 100 µm. Magnification: 20x. Results are expressed as mean ± SEM of one independent experiment, *n = *5 per group in each experiment and were analysed by Two-way ANOVA with multiple comparisons. EAE—Experimental autoimmune encephalomyelitis; WM—White matter; GM—Gray matter; P—Plaque, PP—Periplaque; NAWM – Normal appearing white matter; MBP – Myelin basic protein; Iba1—Ionized calcium binding adaptor molecule 1; GFAP—Glial fibrillary acidic protein; GL– Granular layer; ML – Molecular layer. $ *p < *0.05 vs. 3-month-old EAE mice; $$$ *p < *0.001 vs. 6-month-old EAE mice; ### *p < *0.001 vs. 6-month-old EAE mice

**Fig. 3:**
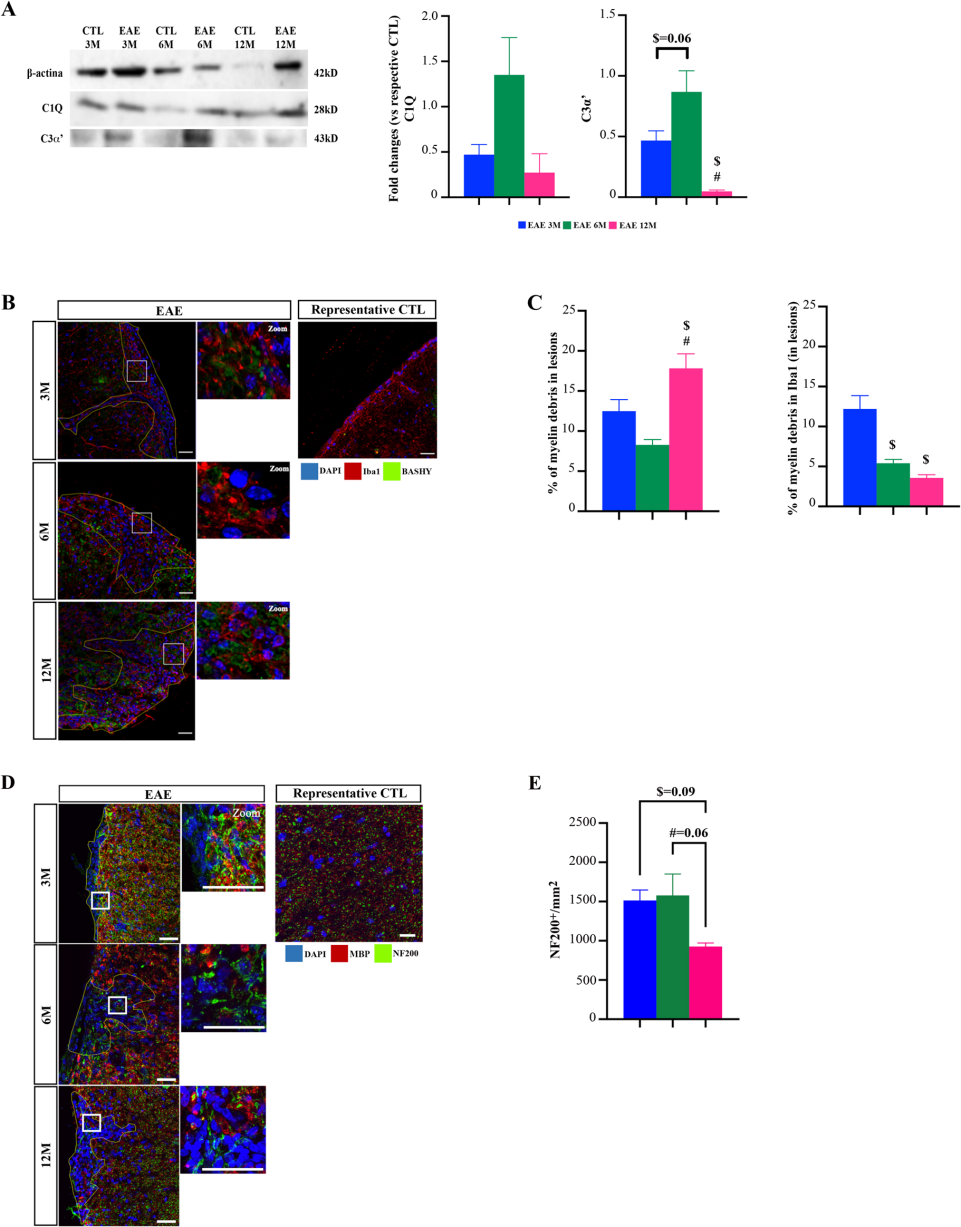
Middle-aged experimental autoimmune encephalomyelitis (EAE) mice showed a decreased complement response and reduced myelin debris phagocytosis by microglia. Female C57BL/6 mice at 3-, 6- and 12-month-old (M) were induced with EAE by MOG_35–55_ immunization and followed for 23 days post-EAE induction; lumbar and thoracic portions of spinal cords were collected at the end of experiment and processed for immunohistochemistry and western blot, respectively. **A** Representative western blot images and relative densitometric quantification of C1q and C3α’ expression. Results were normalized to endogenous β-actin and fold changes for each experimental groups were normalized to young control mice (3-month-old) followed by normalization of the EAE induced mice to their respective control group. **B** Representative images of spinal cords immunostained to identify microglia (Iba1, red) and myelin debris (BASHY probe, green). **C** Graph bars show the percentage of myelin debris in total white matter lesioned area and colocalization of myelin debris with microglia within the lesioned WM area. **D** Representative images of spinal cord lesioned areas delineated by increased nuclei (DAPI—blue staining) without myelin (MBP—red staining). Neurofilaments were identified by NF200^+^ cells (green) staining. White squares represent a zoom section within lesions. **E** Graph bar represents the number of NF200^+^ cells counted in lesioned area. Scale bar: 100 µm. Magnification: 40x. Results are expressed as mean ± SEM of one independent experiment, *n = *5 per group for each experiment. Results were analysed by Two-way ANOVA with multiple comparisons. EAE—Experimental autoimmune encephalomyelitis; CTL—Control; Iba1—Ionized calcium binding adaptor molecule 1; MBP – Myelin basic protein; NF200 – Neurofilament 200. $ *p < *0.05 *vs.* 3-month-old EAE mice; # *p < *0.05 *vs.* 6-month-old EAE mice

Twelve-month-old EAE mice had a higher percentage of microglia reactivity in the plaque lesion, identified by Iba1 immunostaining, compared to 3- and 6-month-old EAE mice (Fig. 2A, *p < *0.001 and Supplementary Fig. 2B). Given the recent findings highlighting the existence of smoldering plaques characterized by microglial activation and slow expansion of pre-existing plaques in progressive MS (Frischer et al. 2015), we also evaluated microglia in the surrounding areas indicated as PP and NAWM. However, in this case no changes were observed between the different EAE mouse age groups. We also observed a significant increase in astrocyte reactivity, identified by GFAP immunostaining, in 12-month-old EAE mice compared to 3-month-old EAE mice in the plaque lesions (Fig. 2A, *p < *0.05 and Supplementary Fig. 2B), again with no significant differences in PP and NAWM areas. Since our previous data indicate the presence of demyelinating lesions in the cerebellum, we also evaluated cerebellar glial reactivity. However, we did not find significant differences between age groups, as shown in Fig. 2B and in Supplementary Fig. 3B. Age-matched control groups showed no lesions and consequently no glial reactivity, as expected, as depicted in Fig. 2A and B, and in Supplementary Fig. 2A and Fig. 3A.

Taken together, our data suggest that, although there are fewer demyelinating lesions in the spinal cord at advanced ages (Fig. 1A), these lesions have increased glial reactivity that may contribute to the worse disease phenotype as previously shown (Ribeiro et al. 2022).

### Middle-aged EAE Mice Show Impaired Complement Response and Myelin Debris Clearance by Microglia

In mouse models of MS, specifically the EAE model, induction of disease activates microglia and astrocytes resulting in the modulation of inflammatory and homeostatic pathways (Ponath et al. 2018; Acharjee et al. 2021). Since we observed age-associated changes only in spinal cord demyelinated lesions (Fig. 1 and 2), we further studied the role of age in EAE molecular pathogenesis first by investigating spinal cord gene expression profile concerning EAE pathogenic processes. At the end of each experiment (23 dpi), thoracic spinal cords were isolated, and the gene content was analyzed by qRT-PCR. Specifically, we evaluated a panel of genes involved in the innate immune system, the antioxidant system, leukocyte adhesion, the nuclear factor kappa B (NF-kB) pathway, cholesterol and vitamin D pathways, the microglia sensome system, the complement system, phagocytosis, inflammasome activation, antigen presentation, cytokine regulation, inflammation associated cytokines (including T cell-associated cytokines) and A1 astrocytic genes (Supplementary Fig. 4A).

Regarding astrocytic genes, we observed that 12-month-old EAE mice overexpressed A1 reactive genes – *H2T23* and *GBP2* (*p < *0.05 vs. 3-month-old EAE mice), that may accompany the increased astrocytic reactivity in demyelinated lesions (Fig. 2A). As previously described, astrocytes can be neurotoxically activated by activated microglia (Liddelow et al. 2017), we next sought to understand the pathological mechanisms in microglia that could contribute to the worse disease outcomes observed in 12-month-old EAE-mice. Interestingly, we observed a significant downregulation of regulatory microglia genes essential for its activation and phagocytosis in 12-month-old EAE mice (Supplementary Fig. 4A). Specifically, we found that these EAE mice had a significantly lower expression of *C1QA*, *B* and *C* compared with 3- and 6-month-old EAE mice (Supplementary Fig. 4A*, p < *0.05). This was accompanied by reduction of complement protein expression analysis (C1q and C3α) (Fig. 3A), suggesting that 12-month-old EAE mice have a reduced complement system response. Knowing that impaired complement activation impacts myelin debris clearance by microglia, which may contribute to the failure of the remyelination process (Borucki et al. 2020), we next evaluated microglia phagocytic ability. Indeed, in the gene expression profile data we observed that 12-month-old EAE mice showed a significant downregulation of *triggering receptor expressed on myeloid cells 2* (*TREM2*) and *MER proto-oncogene, tyrosine kinase (MERTK)* when compared with both 3- and 6-month-old EAE mice (Supplementary Fig. 4A, *p < *0.05). So, we next investigated myelin debris clearance by microglia by immunostaining of myelin debris using an established BASHY dye probe (Pinto et al. 2021). As observed in Fig. 3B and in Supplementary Fig. 5A, 12-month-old EAE mice had significantly higher myelin debris accumulation in spinal cord lesions when compared with both 3- and 6-month-old EAE mice (Fig. 3C, *p < *0.05). Thus, 12- and even 6-month-old EAE mice present with reduced colocalization of BASHY-myelin debris with microglia staining, when compared with 3-month-old EAE mice (Fig. 3C, *p < *0.05). This is indicative of reduced microglia ability to phagocytose and myelin debris from plaque.

As axonal loss is recognized as a major contributor to disease progression (Constantinescu et al. 2011) we sought to understand whether age could contribute to axonal degeneration in EAE mice. Therefore, we assessed neurofilament (NF) 200 staining within spinal cord normal appearing white matter (NAWM, Supplementary Fig. 6C) or within white matter lesions (Fig. 3D and Supplementary Fig. 6A) (Kaneko et al. 2006). Although not significant, we observed a tendency for a decrease of NF200 + axons in 12-month-old EAE mice compared with both 3- and 6-month-old EAE mice in spinal cord lesions (Fig. 3E). Interestingly, we also observed that EAE markedly reduced NF200 staining in the NAWM compared to the respective age-matched control group (Supplementary Fig. 6C, *p < *0.001 3-month-old CTL vs 3-month-old EAE mice and *p < *0.01 12-month-old CTL vs 12-month-old EAE mice), suggesting that EAE by itself may impact the overall spinal cord white matter.

Together, our data demonstrates that the dysfunctional complement system may impact the ability of microglia to phagocytose myelin debris resulting from EAE-induced demyelination, while in parallel the is a reduction of maintained axons within the lesions. This may contribute to failure of the remyelination process in 12-month-old EAE mice and help explain the more severe disease phenotype seen in these animals (Ribeiro et al. 2022).

### Middle-aged EAE Mice Show an Increased Infiltration of CD4 and CD8 T cells in Spinal Lesions and Altered Regulatory T cell Profile at the Spleen

**Fig. 4:**
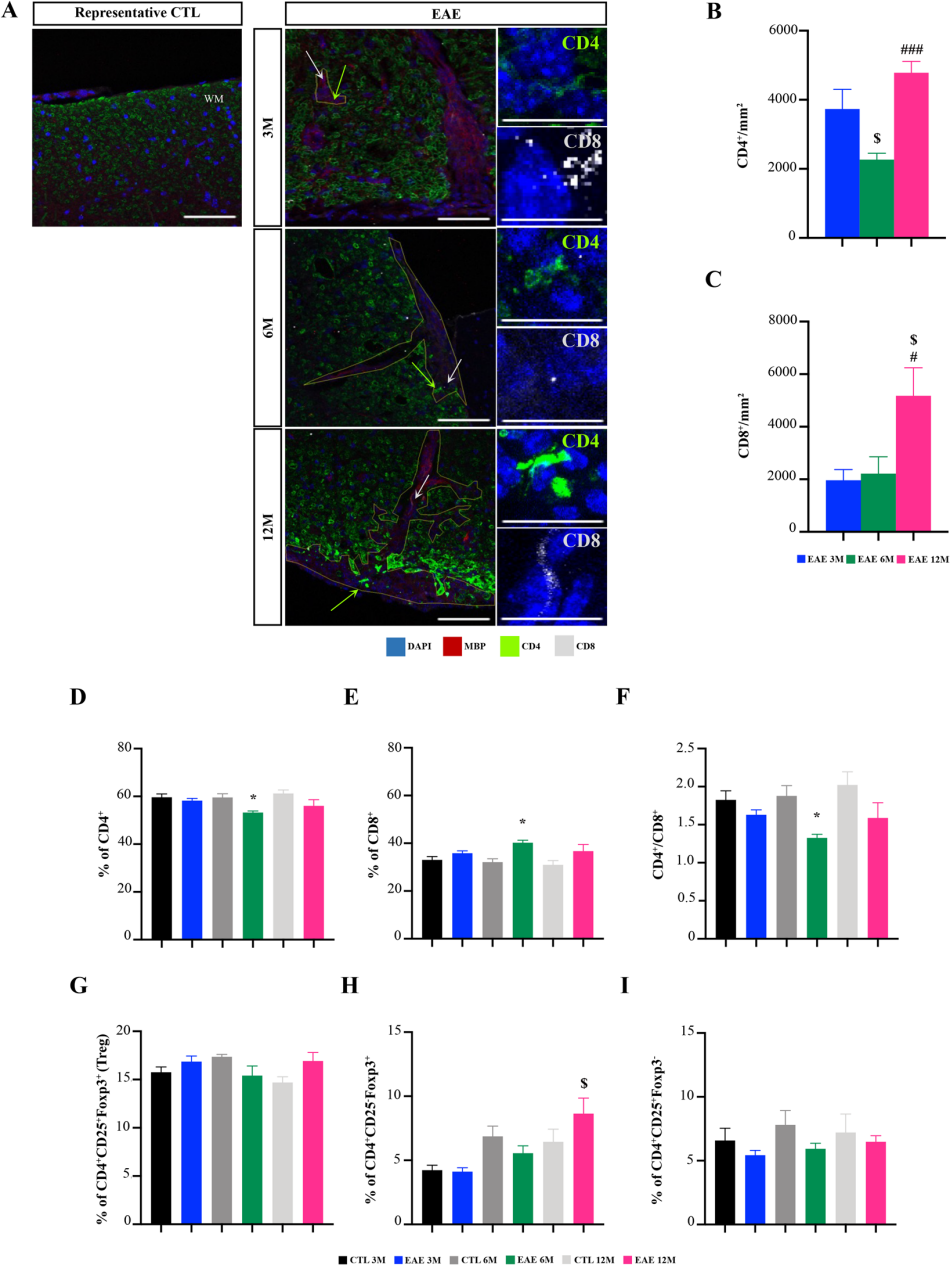
Middle-aged experimental autoimmune encephalomyelitis (EAE) mice show increased T cell infiltrates at the spinal cord and altered peripheral immune profile. Female C57BL/6 mice at 3-, 6- and 12-month-old were induced with EAE by MOG_35–55_ immunization and followed for 23 days post-EAE induction; spinal cord and spleen were collected at the end of the experiment and processed for immunohistochemistry and flow cytometry, respectively. **A** Representative images of CD4 and CD8 T cells staining in demyelinated lesions in spinal cord. Scale bar: 100 µm. Magnification: 40x. Green and grey arrows represent CD4 and CD8 T cells, respectively. Graph bars represent the number of CD4^+^ (green) (**B**) and CD8^+^ (white) (**C**) T cells counted in lesioned area. T cell subsets in the spleen were evaluated by flow cytometry and graph bars represent the percentage of (**D**) CD4^+^ and (**E**) CD8^+^ T cells within parent population and the (**F**) CD4/CD8 ratio; and the percentage of (**G**) regulatory T cells (CD4^+^/CD25^+^/Foxp3^+^), (**H**) CD4^+^/CD25^−^/Foxp3^+^ and (**I**) CD4^+^/CD25^+^/Foxp3^−^ cells within parent population. Sorting strategy is described in Supplementary Fig. 1. Results are expressed as mean ± SEM of one independent experiment, *n = *5 per group for each experiment. Results were analysed by Two-way ANOVA with multiple comparisons and by unpaired two-tailed Student’s t-test between EAE and respective control. EAE—Experimental autoimmune encephalomyelitis; CTL – Control; WM – White matter. $ *p < *0.05 *vs.* 3-month-old EAE mice; # *p < *0.05 *vs.* 6-month-old EAE mice and ### *p < *0.001 *vs.* 6-month-old EAE mice; * *p < *0.05 *vs.* respective control

T lymphocytes (including T helper (Th) 1 and Th17) play a central role in the pathogenesis of MS and in EAE, with both CD4 and CD8 T cells present in MS lesions (van Langelaar et al. 2020). In CNS, these T lymphocytes have an active role in inflammatory cytokine production contributing to the inflammatory environment and disease pathology (Goverman 2009). In contrast, regulatory T cells (Treg), another cell subgroup of CD4^+^ T cells, have an active role in the resolution of inflammation by promoting an anti-inflammatory environment at the site of inflammation (Dargahi et al. 2017). Therefore, we examined CD4 and CD8 T cell infiltration within spinal cord lesions (Fig. 4A). Interestingly, as shown in Fig. 4B, 12-month-old EAE mice displayed higher amounts of CD4 T cells compared to 6-month-old EAE mice (*p < *0.001), although these 6-month-old EAE mice showed a reduced CD4 T cell infiltration when compared to 3-month-old EAE mice (*p < *0.05). With respect to CD8 T cells (Fig. 4C), 12-month-old EAE mice exhibited a marked increase in infiltration in spinal cord lesions when compared with both 3- and 6-month-old EAE mice (*p < *0.05).

To further corroborate these results, we next evaluated T cell populations at the experimental endpoint (23 dpi) in the periphery, namely at the spleen, by flow cytometry. A reduction in CD4^+^ T cells was found in 6-month-old EAE mice when compared to respective controls, while the percentage of CD8^+^ T cells was significantly increased in 6-month-old EAE mice (Fig. 4E, *p < *0.05). Although non-significant, similar trends were found in the 3- and more evident in the 12-month-old EAE mice when compared to their respective controls, both for CD4^+^ and CD8^+^ T cells (Fig. 4D-E). These changes resulted in a decreased CD4^+^/CD8^+^ ratio in the EAE groups when compared to their respective control groups (Fig. 4F). This was statistically significant for the 6-month-old EAE mice when compared to their respective controls (*p < *0.05). These data suggest that T cell populations are affected by age, with the most cytotoxic population seen at 6 months of age.

We next assessed Treg cells by comparing the expression of their specific marker – Foxp3 – as described previously (Nishioka et al. 2006). Our results showed similar percentages of classical activated Treg cells (CD4^+^CD25^+^Foxp3^+^) in 3-, 6- and 12-month-old groups (Fig. 4G). However, age significantly increased the percentage of CD4^+^CD25^−^Foxp3^+^ cells as shown by a significant increase of this population in 12-month-old EAE mice when compared to 3-month-old EAE mice (Fig. 4H, *p < *0.05). Curiously, this T cell population has been reported to have a hyperresponsiveness to stimuli (Nishioka et al. 2006), with lower regulatory and suppressive properties (O’Gorman et al. 2009; Chavele and Ehrenstein 2011), being even reported as exhausted Treg cells (Zohouri et al. 2021).

The upregulation of T cell-associated cytokines may reflect the increased number of CD4 T cells, and especially CD8 T cells in spinal cord lesions. Indeed, in our gene expression profile data, we showed that 12-month-old EAE mice exhibited upregulation of pro-inflammatory cytokines Interferon (*IFN*)*-γ* and Interleukin (*IL*)*−17*, accompanied by downregulation of the anti-inflammatory cytokine *IL-10* when compared to both 3- and 6-month-old EAE mice (Supplementary Fig. 4A, *p < *0.05). This may contribute to the spinal cord inflammatory environment in middle-aged EAE mice.

Overall, these results suggest that a more cytotoxic effect of CD8 T cells, and not only CD4 T cells as previously described (Atkinson et al. 2022), in 12-month-old EAE mice may contribute to exacerbation of the EAE pathogenesis. Moreover, EAE induction together with age seems to alter the T cell equilibrium, leading to increased number of cytotoxic cells, and affecting Treg cells. This indicates that 12-month-old EAE mice may have a less-suppressive and less-tolerant EAE pathogenic phenotype than younger EAE animals.

### Neuroinflammation rescue in EAE S100B KO Mice Reduces EAE Clinical Score and FI Mostly in Younger EAE Mice

S100B has been described as one important inflammatory marker in MS (Petzold 2002; Barateiro et al. 2016). Based on recent data, S100B was found to be increased in spinal cord of 8–12 weeks EAE mice compared to controls and, the depletion of S100B partially protected against EAE-associated paralysis, which was accompanied by reduced glial reactivity and inflammation (Barros et al. 2022). Additionally, at the protein level, there is a tendency for higher S100B expression in EAE mice at 6- and 12- month-old compared to 3-month-old EAE mice at the chronic phase (Supplementary Fig. 7). However, the role of S100B ablation in EAE at different ages has not yet been explored. Here, we induced female S100B KO mice with EAE at three different ages: 3-, 6- and 12-month-old of age (young S100B KO, adult S100B KO and middle-aged S100B KO, respectively) and followed them for 23 days. Age-matched S100B KO control groups without MOG_35–55_ induction were used.

**Fig. 5:**
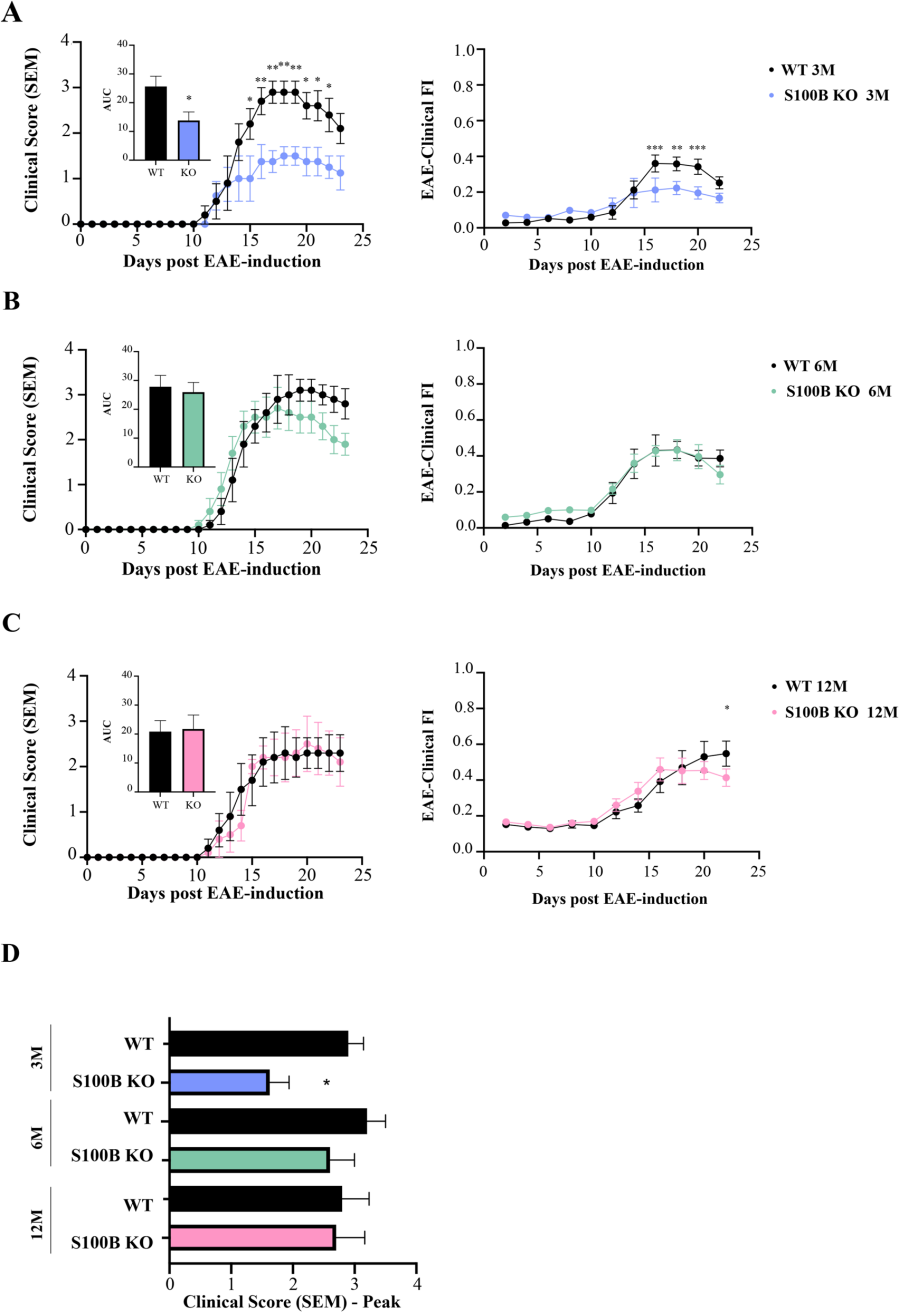
Experimental autoimmune encephalomyelitis (EAE) S100B KO mice showed lower frailty index (FI) and clinical scores (CS) compared to EAE WT mice of the same age. Female S100B KO and WT C57BL/6 mice at 3-, 6- and 12-month-old were induced with EAE by MOG35-55 immunization and followed for 23 days post-EAE induction. The 5-point clinical score and the 34-item EAE-Clinical FI scales were used to quantify overall health. Clinical scores were assessed daily, and he EAE-Clinical FI scores were assessed every two days in (**A**) 3-month-old, (**B**) 6-month-old and (**C**) 12-month-old EAE S100B KO mice and compared to the respective EAE WT mice of the same age (black line). The AUC in each panel was measured for individual mice in each group. **D** Graph bars indicate the peak clinical disease score of EAE WT and EAE S100B KO mice. Results are expressed as mean ± SEM of one independent experiment, *n = *4–5 per group for each experiment and analysed by Two-way ANOVA with multiple comparisons. EAE—Experimental autoimmune encephalomyelitis; FI – Frailty Index; CS – Clinical Score; AUC – Area under the curve. * *p < *0.05, ** *p < *0.01, *** *p < *0.001 vs. respective EAE WT from the same age

First, to assess overall health, both the EAE-Clinical FI scale (Ribeiro et al. 2022) and the traditional 5-point clinical score scale (Berard et al. 2010; Robinson et al. 2014) were used and the S100B KO mice phenotype was compared to the WT mouse phenotype at the same age (Fig. 5, Supplementary Table 2 and 3). Depletion of S100B in 3-month-old EAE S100B KO mice resulted in significantly lower traditional clinical score when compared to age-matched EAE WT mice (Fig. 5A, *p* < 0.01), being more evident at the peak of disease around 14–19 dpi (Fig. 5D, *p < *0.05). The same effect was seen in terms of the EAE-Clinical FI (Fig. 5A), where 3-month-old EAE S100B KO mice displayed lower scores (maximum 1.5 at 17 dpi) compared to age-matched EAE WT mice (maximum 3 at 17 dpi), with a marked prevention of paralysis events (Supplementary Table 3). By contrast, 6-month-old EAE S100B KO mice showed no significant changes in EAE clinical score and in EAE-Clinical FI (Fig. 5B) when compared to EAE WT. Nevertheless, there was a declining curve for the EAE clinical score from 19 dpi onwards, showing a partial recovery, and a modest decrease in the EAE-Clinical FI at the end of the experiment (23 dpi) (Fig. 5B). Lastly, 12-month-old EAE S100B KO mice had similar EAE clinical scores compared to the respective EAE WT animals, but the EAE-Clinical FI scores were significantly lower at the end of experiment (23 dpi) when compared to aged-matched EAE WT mice (Fig. 5C, *p < *0.05). These data agree with previous findings (Ribeiro et al. 2022), where middle-aged EAE mice showed an atypical EAE phenotype with features beyond motor symptoms. In fact, with the EAE-Clinical FI it is possible to assess other systems. In this case, we observed that 12-month-old EAE WT (Supplementary Table 2) mice had higher scores in physical condition, neuromusculoskeletal/sensorimotor reflex, weakness and coordination systems that were partially reduced in EAE S100B KO mice from 20 dpi onwards (Supplementary Table 3).

These results suggest that depletion of S100B could ameliorate EAE motor symptoms, an effect which was more pronounced at younger ages (3-month-old). In older EAE mice, S100B attenuated additional symptoms detected by the EAE-Clinical FI. This may suggest that S100B, and therefore S100B-associated neuroinflammation, plays a more central role in EAE motor symptomatology at younger ages, while contributing to other deficits at later ages.

### Depletion of S100B Ameliorates EAE-like CNS Pathology, Partially Restores Myelin Clearance and Protects from Axonal Degeneration in Middle-aged Mice

As described previously, toxic levels of S100B affect demyelination and increase gliosis (Barateiro et al. 2016; Santos et al. 2018). Therefore, here we examined the effects of S100B depletion in EAE mice at different ages to understand how EAE S100B KO mice could have a less severe EAE phenotype.

**Fig. 6:**
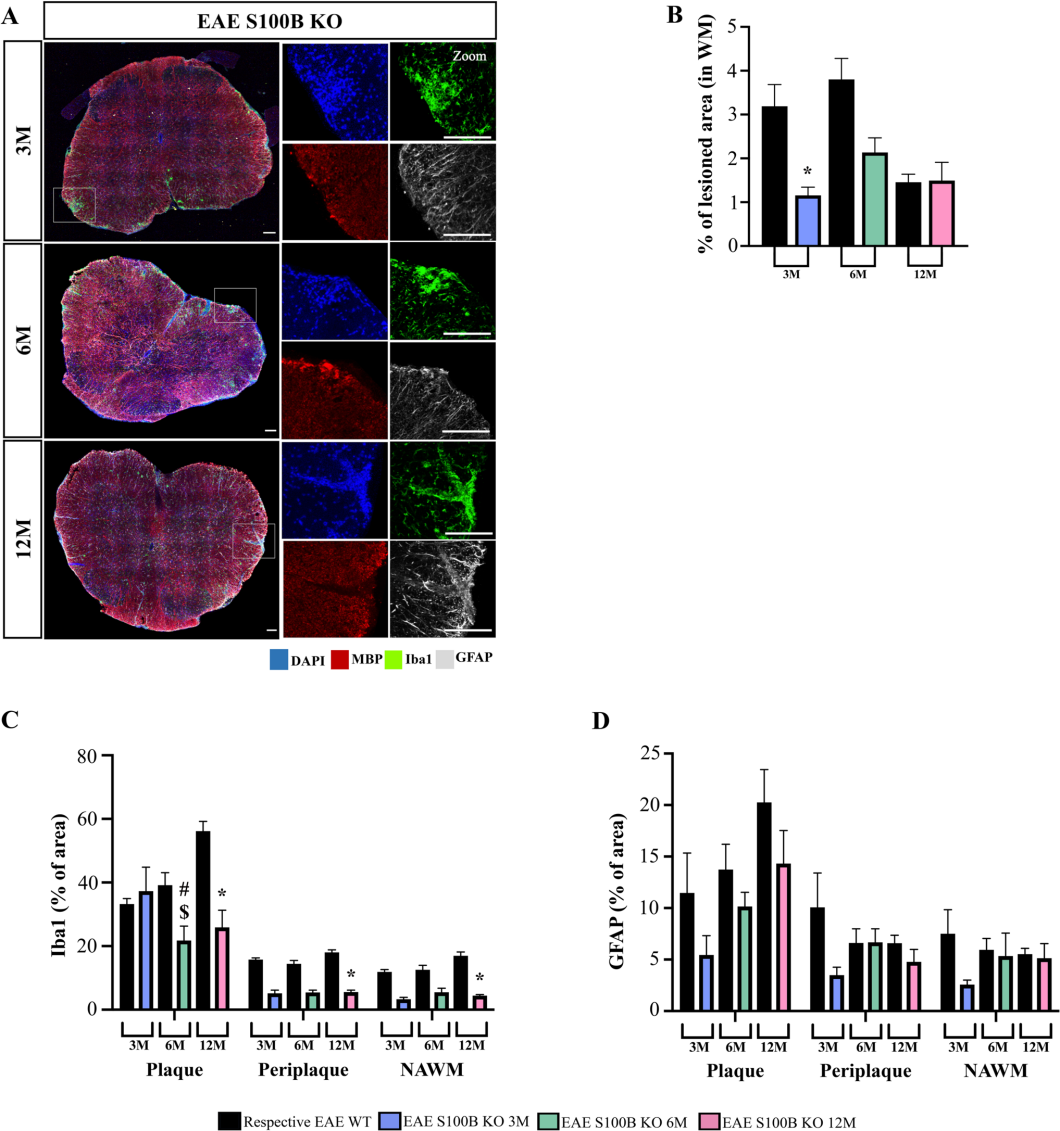
The depletion of S100B ameliorates experimental autoimmune encephalomyelitis (EAE) like pathology. Female S100B KO and WT C57BL/6 mice at 3-, 6- and 12-month-old were induced with EAE by MOG_35–55_ immunization and followed for 23 days post-EAE induction. Lumbar spinal cord was isolated at the end of the experiment and processed for immunohistochemistry to identify myelin (MBP), microglia (Iba1) and astrocytes (GFAP). **A** Representative images of spinal cord sections showing lesioned area, delineated by increased DAPI (blue) without MBP (red) staining with Iba1 (green) and GFAP (white) expression. Scale bar: 100 µm. Magnification: 20x. White square represents a delineated lesion. **B** Percentage of lesioned area was calculated in relation to total area of white matter (WM) and the percentage of (**C**) microglia and (**D**) astrocytes was measured in plaque (P), periplaque (PP) and normal appearing white matter (NAWM). Results are expressed as mean ± SEM of one independent experiment, *n = *4–5 per group for each experiment and were analysed by Two-way ANOVA with multiple comparisons and by unpaired two-tailed Student’s t-test between EAE WT and KO mice. EAE—Experimental autoimmune encephalomyelitis; WM – White matter; P – Plaque, PP – Periplaque; NAWM – Normal appearing white matter; WM – White matter; Iba1—Ionized calcium binding adaptor molecule 1; GFAP—Glial fibrillary acidic protein. * *p < *0.05 vs. respective EAE WT mice; $ *p < *0.05 vs. 3-month-old EAE KO mice; # *p < *0.05 vs. 6-month-old EAE KO mice

S100B depletion resulted in a significant decrease in the percentage of lesioned area in 3-month-old EAE S100B KO mice compared to age-matched EAE WT mice (Fig. 6A-B and Fig. 2A respectively, *p < *0.05). There was a similar trend for 6-month-old mice, although not significant, but there were no differences in 12-month-old mice, in accordance with the symptomatology accessed by the EAE clinical scores and the EAE-Clinical FI observed for the EAE mice at each age group.

We next assessed glial activity following Iba1 and GFAP staining (Fig. 6A, C and D, respectively). S100B depletion significantly reduced the percentage of microglia in spinal cord lesions in both 6- and 12-month-old EAE S100B KO mice when compared to age-matched EAE WT mice (*p < *0.05). Interestingly, this reduction was sustained in PP and NAWM with a significant impact in 12-month-old EAE S100B KO mice (Fig. 6C, *p < *0.05). Regarding astrocyte reactivity (Fig. 6D), there was a slight reduction in the EAE S100B KO mice when compared with age-matched EAE WT groups in plaque lesions for all age cohorts, although not this was not significant. The same tendency was observed for the PP and NAWM areas, but in this case mostly for the 3-month-old EAE S100B KO mice.

**Fig. 7:**
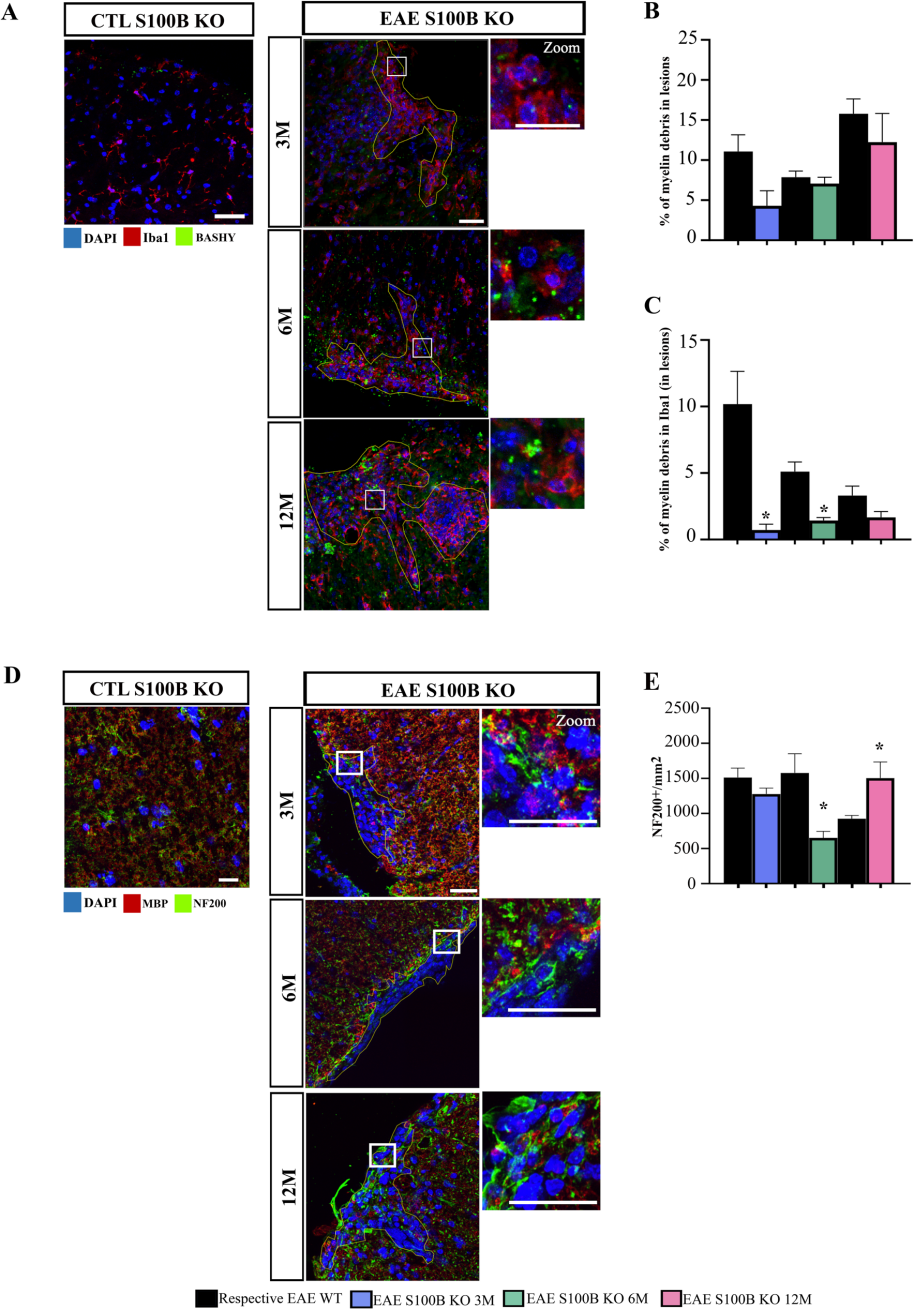
S100B ablation partially restores myelin clearance and appears to protect axonal degeneration in middle-aged mice. Female S100B KO and WT C57BL/6 mice at 3-, 6- and 12-month-old were induced with EAE by MOG_35–55_ immunization and followed for 23 days post-EAE induction. Lumbar spinal cord was isolated at the end of the experiment and processed for immunohistochemistry. **A** Representative images of spinal cords immunostained to identify microglia (Iba1, red) and myelin debris (BASHY probe, green). **B** Graph bars show the percentage of myelin debris in total WM lesioned area and (**C**) colocalization of myelin debris with microglia within the lesioned area. **D** Representative images of spinal cord lesioned areas delineated by increased nuclei (DAPI—blue staining) without myelin (MBP—red staining). Neurofilaments were identified by NF200^+^ cells (green) staining. White squares represent a zoom section within lesions. **E** Graph bar represents the number of NF200^+^ cells counted in lesioned area. Scale bar: 100 µm. Magnification: 40x. Results are expressed as mean ± SEM of one independent experiment, *n = *4–5 per group for each experiment and were analysed by Two-way ANOVA with multiple comparisons and by unpaired two-tailed Student’s t-test between EAE WT and KO mice. EAE—Experimental autoimmune encephalomyelitis; CTL—Control; Iba1—Ionized calcium binding adaptor molecule 1; MBP – Myelin basic protein; NF200 – Neurofilament 200 * *p < *0.05 vs. respective EAE WT mice

Based in these results we also evaluated our gene expression panel in the EAE S100B KO spinal cord mice (Supplementary Fig. 4B). In terms of inflammatory and functional genes associated with microglia signature, 3- and 6-month-old EAE S100B KO mice showed a downregulation of *translocator protein* (*TSPO*) (*p < *0.05) when compared to age-matched EAE WT mice, while only 3-month-old EAE S100B KO showed a downregulation of the *major histocompatibility complex* (*MHC*)*-II* genes (*p < *0.05) when compared to age-matched EAE WT groups. Interestingly, concerning microglia phagocytic genes that were impaired in 12-month-old EAE WT mice, we clearly observed an upregulation of both *MERTK* and *TREM2* in S100B KO mice at this age (*p < *0.05), restoring them to values observed in younger animals. Curiously, when we next assessed myelin debris clearance at lesion site using the BASHY probe (Fig. 7A and Supplementary Fig. 5B), there was a trend to reduce myelin debris accumulation at the plaque for all age cohorts (Fig. 7B). However, there was a marked reduction of microglia myelin phagocytosis (Fig. 7C), and this was significant for 3- and 6-month-old EAE S100B KO mice, which may suggest a faster myelin turnover in these S100B KO mice.

We then assess axonal degeneration in the S100B KO model to understand whether the S100B ablation could contribute to axonal protection and consequent improvement of disease course (Fig. 7D and Supplementary Fig. 6B). Interestingly, we observed that S100B depletion increase NF200 + axons in demyelinated lesions in 12-month-old EAE S100B KO mice (Fig. 7E, *p < *0.05 vs respective EAE WT mice). We also observed that CTL S100B KO mice also exhibited an increase in NF200 positive cells compared to the respective CTL WT mice (Supplementary Fig. 6C). This result suggests that the protection of axonal degeneration may contribute for the improvement of the disease course as described in Fig. 5.

Overall, these results indicate that S100B depletion has a critical effect in ameliorating neuroinflammation, with less glial reactivity, less activation of inflammatory gene markers and improvement of NF200 + axons in 12-month animals. Moreover, microglia phagocytic dysfunction seems to be shifted to a more effective capacity revealing that depletion of S100B could be beneficial to the process of remyelination.

### Immune Response in the Periphery Shifts to A More Regulatory Phenotype in the Middle-aged EAE S100B KO Mice

**Fig. 8:**
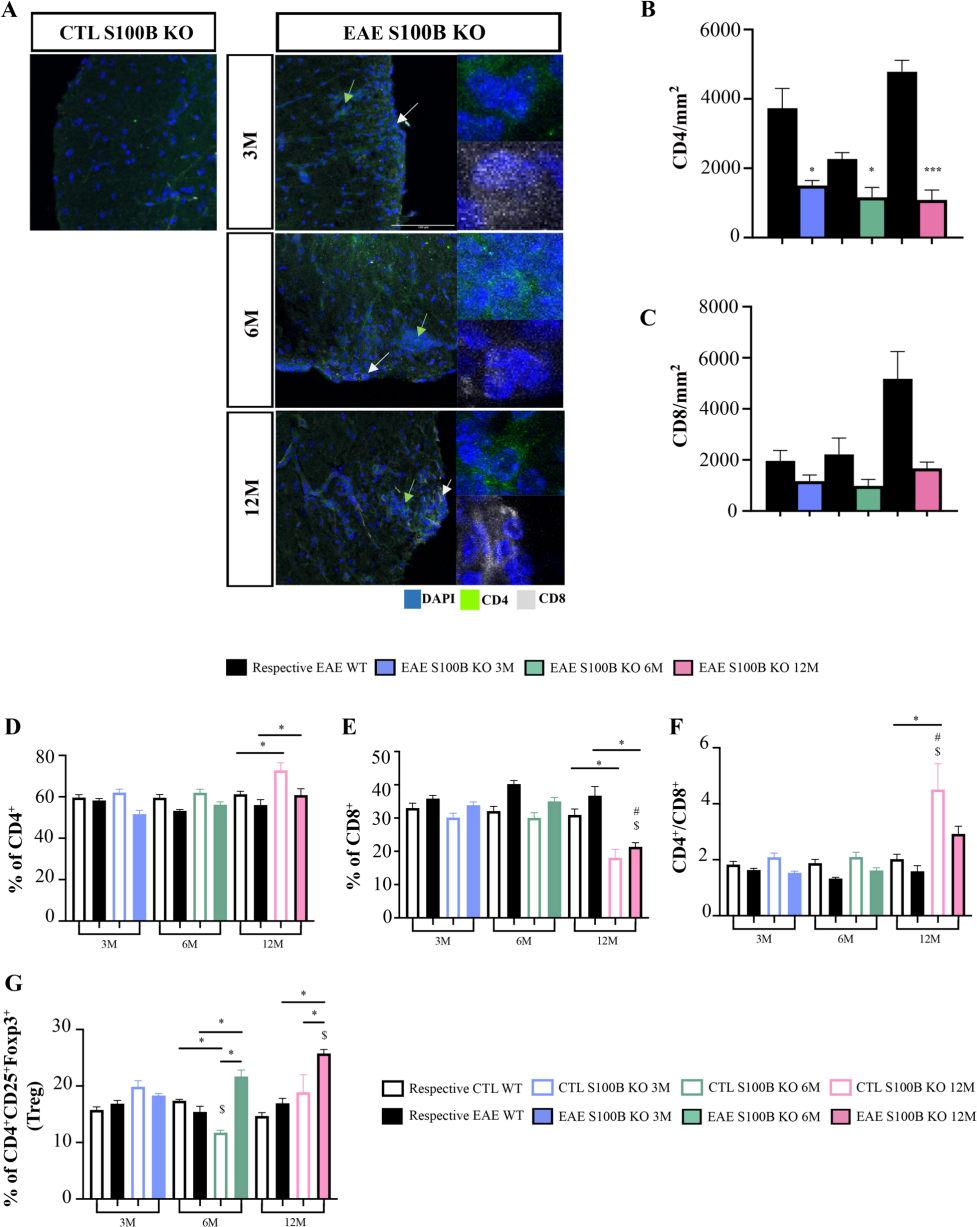
The depletion of S100B led to a more regulatory immune response in middle-aged experimental autoimmune encephalomyelitis (EAE) S100B KO mice. Female S100B KO and WT C57BL/6 mice at 3-, 6- and 12-month-old were induced with EAE by MOG_35–55_ immunization and followed for 23 days post-EAE induction. Spinal cords and spleens were collected at the end of the experiment and processed for immunohistochemistry and flow cytometry, respectively. **A** Representative images of CD4 (green) and CD8 (white) staining in demyelinated lesions in spinal cord. Scale bar: 100 µm. Magnification: 40x. Green and grey arrows represent CD4 and CD8 cells, respectively. **B** Graph bars represent the number of CD4^+^ and (**C**) CD8^+^ cells counted in lesioned area. T cell subsets in the spleen were evaluated by flow cytometry and graph bars represent the percentage of (**D**) CD4, (**E**) CD8, (**F**) the CD4/CD8 ratio and (**G**) percentage of regulatory T cells within parent population. Results are expressed as mean ± SEM of one independent experiment, *n = *4–5 per group for each experiment and were analysed by Two-way ANOVA with multiple comparisons. Sorting strategy is described in Supplementary Fig. 1. EAE—Experimental autoimmune encephalomyelitis; Treg – regulatory T cells. * *p < *0.05 and *** *p < *0.001 vs. respective control or EAE WT mice; $ *p < *0.05 vs. 3-month-old EAE KO mice; # *p < *0.05 vs. 6-month-old EAE KO mice

In a previous study we showed that S100B blockade improves T cell regulatory responses in 3-month-old EAE WT mice (Barros et al. 2022), so we next assessed whether CD4 and/or CD8 T cell infiltration in spinal cord could be altered by S100B depletion (Fig. 8A-C). As shown in Fig. 8B, there is a marked reduction in the number of infiltrating CD4 T cells in EAE S100B KO mice and this was significant for 3-, 6- (*p < *0.05) and 12-month-old (*p < *0.001) when compared to age-matched EAE WT mice. A similar prevention is observed for infiltration of CD8 T cells (Fig. 8C), with a significant impact in 12-month-old EAE S100B KO mice when compared to age-matched EAE WT mice (*p < *0.05). Curiously, when evaluating the expression of T cell associated cytokines in our gene expression data (Supplementary Fig. 4B), we observed that *IFN-γ* was slightly downregulated in 3-month-old EAE S100B KO mice but upregulated in 6-month-old EAE S100B KO mice, while no differences were observed in 12-month-old EAE S100B KO mice when compared to age-matched EAE WT mice. Moreover, we observed a significant upregulation of *IL-17* in both 6- and 12-month-old EAE S100B KO mice when compared to age-matched EAE WT mice (*p < *0.05). These results were accompanied by upregulation of *IL-10* with a significant impact in 6-month-old EAE S100B KO mice when compared to aged-matched EAE WT mice (*p < *0.05).

Finally, we evaluated the peripheral immune response upon S100B depletion (Fig. 8D-G). Flow cytometry results revealed major alterations in immune cell profiles in 6- and 12-month-old S100 KO mice. Indeed, 12-month-old control S100B KO mice showed a significant increase in the percentage of CD4^+^ T cells (*p < *0.05, Fig. 8D) in contrast to the reduction in CD8^+^ T cells (*p < *0.05, Fig. 8E) when compared to age-matched control WT group. This may account for the observed increase in CD4^+^ T cells (*p < *0.05, Fig. 8D) and decrease in CD8^+^ T cells (*p < *0.05, Fig. 8E) when 12-month-old EAE S100B KO mice were compared to age-matched EAE WT group. These changes resulted in a tendency to increase CD4^+^/CD8^+^ ratio in 12-month-old S100 KO mice compared to age-matched EAE WT group (Fig. 8F). Most interestingly, there was a significant increase in the percentage of CD4^+^CD25^+^Foxp3^+^ regulatory T cells in 6- and 12-month-old EAE S100B KO when compared to age-matched EAE WT groups (Fig. 8G, *p < *0.05), or even when 12-month-old EAE S100B KO mice were compared to 3-month-old EAE S100B KO mice (Fig. 8G, *p < *0.05).

In summary, these data indicate that S100B depletion has a critical effect in ameliorating neuroinflammation, enabling reduced CD8^+^ T cell cytotoxic mediated responses, in parallel with an enhanced Treg cell response at older ages.

### S100B Ablation Brought Closer Phenotypical Characteristics Between 3-month-old EAE S100B KO and Non-EAE Controls

Using principal component analysis (PCA), we plotted the previously analyzed variables to see whether they could distinguish our experimental groups in terms of mouse age, EAE induction and S100B ablation. Supplementary Fig. 8 shows the score plots of PC1 versus PC2. As depicted in the graph, the items compared between WT (Supplementary Fig. 8A and B) and S100BKO (Supplementary Fig. 8E and F) animals (i.e. clinical score, EAE-Clinical FI, demyelinating lesions, glial reactivity, spinal cord gene expression, and immune response at the periphery) could distinguish between the mouse groups. We showed that, in WT mice (Supplementary Fig. 8A and B), control 12-month-old mice are different from the young and adult mice, which could explain why 12-month-old EAE mice have such a distinct phenotype when compared to 3- and 6-month-old EAE mice. Importantly, when we also plotted only the set of genes represented in Supplementary Fig. 4A, we observed that they are the ones that contribute most for the segregation of the groups (Supplementary Fig. 8C and D), once they maintained the same tendency as in Supplementary Fig. 8A. The specific results for each variable are represented in Supplementary Table 4. Interestingly, when we plotted S100B KO mice (Supplementary Fig. 8E and F), we observed that the ablation of S100B led to a more homogeneous population between control S100B KO mice at all ages and led to the 3-month-old EAE S100B KO resembling control mice, indicating that those groups had similar read outs. This is in accordance with reduced EAE pathogenesis. Moreover, when we plotted only the set of genes represented in Supplementary Fig. 4B, we observed a shift between 3- and 6-month-old S100B CTL and S100B EAE mice (Supplementary Fig. 8G and H). The specific results for each variable are represented in Supplementary Table 5.

These data reinforce the idea that mouse age is a predictor of worse outcomes and upon EAE induction, older mice have a more rapid clinical course with more pronounced EAE pathogenesis and less recovery. Moreover, the absence of S100B is beneficial, as it was able to reduce EAE pathogenesis namely in advanced ages.

## Discussion

This study describes, for the first time, the effects of age on in vivo EAE model of MS induced by active immunization, and the effects of S100B ablation on EAE like-pathology at different ages. The EAE model is a well-established model to study MS pathological features and is the most widely used in pre-clinical studies to implement new DMTs (Lassmann and Bradl 2017; Burrows et al. 2019). However, few studies have investigated how EAE is expressed in mice at advanced ages and how the disease can be evaluated clinically (Matejuk et al. 2005; Aharoni et al. 2013; Seo et al. 2015; Peferoen et al. 2016). A key study conducted by Atkinson et al., demonstrated that EAE induced by the adoptive transfer of myelin reactive active Th17 cells in middle-aged mice (10 to 11-month-old) was more severe with a negative impact on neuroinflammation (Atkinson et al. 2022). Here, we used an active immunization model in order to evaluate the activation of myelin specific T cells, rather than study the effect of a specific type of Th1 or Th17 cells. We demonstrate that age is a predictor of worse outcomes in EAE mice resulting in a more severe disease phenotype, microglial dysfunction and alterations in immune responses. Moreover, we show beneficial effects of reducing neuroinflammation by S100B depletion, namely in the pathological hallmarks of EAE at advanced ages. In fact, the depletion of S100B attenuated symptomatology in younger mice, reduced both demyelination and neuroinflammation and shifted immune system responses towards a more regulatory phenotype, which was more pronounced in middle-aged mice.

MS is a neurodegenerative, autoimmune, and demyelinating disorder of the CNS that affects mainly young adults (Dobson and Giovannoni 2019). It is well-established that age is a predictor of increased susceptibility to adverse outcomes resulting in premature death for many disease conditions (Rockwood et al. 2004; Song et al. 2010; Hoogendijk et al. 2019). However, only a few studies have focused on the role of age in the context of MS. Studies have shown that around 0.6 to 1.2% of middle-aged adults develop a late-onset MS (Buscarinu et al. 2022; Capasso et al. 2023) accompanied with worse disease outcomes and poor responses to the available DMTs (Vaughn et al. 2019; Macaron et al. 2023). However, the molecular disabilities that late-onset MS presents and their contribution to disease course have not been investigated. Our group recently published a study focusing on the impact of age on motor disabilities in EAE mice, using a novel FI scale that showed an increased EAE-clinical FI score at advanced ages (Ribeiro et al. 2022). The present study takes a step towards understanding how age impacts EAE progression by assessing the molecular alterations in EAE mice induced at different ages: at 3-month-old (young mice), 6-month-old (adult mice) and 12-month-old (middle-aged mice). Previously it was reported that middle-aged EAE mice induced by adoptive transfer of activated Th17 cells developed a more severe EAE phenotype together with extensive demyelination (Atkinson et al. 2022). Curiously, in our study we found that 12-month middle-aged EAE mice were less affected with respect to the lesioned area in the spinal cord when compared to the other ages, possibly because other immune cells may be responsible for lesion formation in the present EAE model of active immunization. In fact, these results corroborate the atypical phenotype observed in middle-aged EAE mice described previously (Ribeiro et al. 2022).

The higher FI scores in middle-aged mice led us to hypothesize that other CNS regions could contribute to the atypical EAE phenotype. When we analyzed the cerebellum region, already described in the context of MS (Wilkins 2017), we found that the phenotypic observations in middle-aged animals were not due to cerebellar dysfunction. These observations may be related to the fact that more than 80% of MS patients show spinal cord lesions that can be correlated with variation of the expanded disability status scale (EDSS) and the location of such lesions (Eden et al. 2019). For instance, dorsal spinal cord lesions are frequently found in secondary progressive MS. Moreover, high EDSS and short disease activity are also correlated with a high frequency of lesions in dorsal, lateral and central regions of the spinal cord (Eden et al. 2019). Interestingly, severe EDSS affects regions in motor tracts and in sensory tracts (Eden et al. 2019). Therefore, more studies using magnetic resonance imaging techniques should investigate whether middle-aged EAE mice, with severe phenotypical symptoms but a lower percentage of lesioned area, indeed have a greater number of lesions in both motor and sensory tracts, as previously highlighted for human MS (Eden et al. 2019).

We next analyzed glial reactivity, one of the main hallmarks described within spinal cord lesions in many MS models such as the EAE and cuprizone models (Lassmann and Bradl 2017; Filippi et al. 2018). We found that 12-month-old middle-aged EAE mice developed more pronounced glial reactivity, regarding microglial and astrocytic density. In fact, enhanced glial reactivity has been implicated in the context of MS (Schirmer et al. 2021), and it has been discussed in the context of aging and its contribution to aging-associated neurodegenerative disorders (Salas et al. 2020). Glial cells are the first CNS cells to change function with age, by failing in their neuroprotective role (Niraula et al. 2017). Microglial cells exhibit morphological alterations with age, revealing fewer branches and non-uniform tissue distribution (Edler et al. 2021; Yoo and Kwon 2022). In terms of molecular changes in aging, these cells display increased expression of CD68 and MHC-II with exacerbation of inflammatory mediators’ production and lower levels of homeostatic genes that could lead to failure of phagocytic ability as a result of a constantly primed environment (Edler et al. 2021; Atkinson et al. 2022). On the other hand, astrocytes exhibit an increased production of GFAP with age (Clarke et al. 2018). Astrocytes also tend to increase the production of A1 reactive genes with age, which contributes to a more inflammatory environment, blood–brain barrier disruption and neuronal dysfunction (Clarke et al. 2018; Palmer and Ousman 2018; Colón Ortiz and Eroglu 2024). The effects of astrocytes in MS depend on the neuroanatomical region and proximity to demyelinating plaques, thus contributing to the loss of homeostatic functions and gain of neuroinflammation properties (i.e. decrease of cholesterol synthesis and increase of inflammatory genes) (Barmpagiannos et al. 2023; Colón Ortiz and Eroglu 2024). Moreover, higher levels of GFAP^+^ cells might be a consequence of increased levels of reactive microglia. Previous studies demonstrate that reactive astrocytes are induced by activated microglia, which agrees with our results (Liddelow et al. 2017). In the context of MS, microglial and astrocyte dysfunction along with age may potentiate neuroinflammation and impair remyelination contributing to progressive forms of the disease and patients’ disability (Musella et al. 2018; Absinta et al. 2021).

The gene profile of the spinal cord shows a heterogenous variation between EAE mice at different ages. First, we found that complement associated gene – *C1q* – was downregulated in 12-month-old EAE mice and this was linked to downregulation of complement proteins – C1q and C3 – in these mice. The contribution of the complement system to the pathology of EAE is evident by the deposition of complement components and activation products in CNS lesions (Tatomir et al. 2017). Also, the deposition of these products has been found within astrocytes, blood vessels and importantly, within microglia/macrophages (Ingram et al. 2014). Complement dysregulation has been related to the pathogenesis of several autoimmune disorders, such as systemic lupus erythematosus, where it is directly linked to an excess of immunocomplexes contributing to inflammation and the release of autoantigens that trigger an autoimmune response (Ballanti et al. 2013). Microglia, due to their phagocytic capacity, are able to internalize pathogenic particles (i.e. Aβ deposits in Alzheimer’s disease) and C1q interaction enhances this phagocytic capacity (Webster et al. 2000). Interestingly, our results revealed downregulation of *TREM2* and *MERTK* expression in 12-month-old EAE mice accompanied by a reduced capacity for clearance of myelin debris by microglia, which will negatively impact the process of remyelination (Cignarella et al. 2020). Our results agree with previous studies showing that microglia not only become more reactive with age but also exhibit a decline in phagocytic ability contributing to failure of the uptake of extracellular debris and protein aggregates (Edler et al. 2021; Yoo and Kwon 2022; Li et al. 2023). In MS, microglia and macrophages are crucial for the process of remyelination because of their ability to clear cell debris by phagocytic processes (Voß et al. 2012; Pinto and Fernandes 2020). In fact, previous studies using a lysolecithin mouse model demonstrated that the depletion of macrophages reduces remyelination (Kotter 2006). Moreover, TREM2 blockage in an EAE model resulted in disease exacerbation with more CNS inflammation and demyelination (Piccio et al. 2007). By contrast, TREM2 overexpression improved myelin removal in lesion areas facilitating disease recovery (Takahashi et al. 2005). Our data demonstrate that 12-month-old EAE mice displayed a decline in regulatory mechanisms of microglia homeostasis, including complement and phagocytosis alteration, which contributes to the failure of remyelination. In addition to impaired microglial homeostasis, the observed decline in NF200 staining at older ages suggests that age-related changes in the axonal cytoskeleton may further contribute to remyelination failure in the EAE model. Since NF200 is a key component of neurofilaments that maintain axonal integrity (Laser-Azogui et al. 2015), its reduction in demyelinated lesions could indicate increased axonal susceptibility to damage or impaired neuronal support in aging mice. This aligns with previous findings that age-related neuroinflammatory changes, including dysfunctional microglia, can exacerbate axonal degeneration and hinder effective remyelination (Mancini et al. 2023; Kaneko et al 2006). Given that successful remyelination requires both efficient myelin debris clearance and a supportive neuronal environment, the interplay between microglial dysfunction and axonal integrity may play a crucial role in the reduced recovery observed in older EAE mice. Future studies investigating the mechanistic link between NF200 expression, microglial phagocytic deficits, and remyelination efficiency could provide further insights into age-related remyelination failure in MS. Therefore, our results support previous findings that older mice have worse disease outcomes (Matejuk et al. 2005; Ribeiro et al. 2022).

In MS and in EAE, disruption of the blood–brain barrier allows the leakage of infiltrated immune cells from the periphery thus recruiting more inflammatory cells into the CNS, creating a neuroinflammatory environment and augmenting disease severity (Compston and Coles 2008). Moreover, a chronic inflammatory environment is favored in aging by the release of pro-inflammatory cytokines, contributing to the development of age-related diseases (Finger et al. 2022). We found that EAE induction promoted higher expression of inflammatory cytokines and chemokine genes. Concerning pro-inflammatory cytokines, previous studies indicated that the expression of these factors was significantly increased upon EAE induction (Jahan‐Abad et al. 2020), and our results showed that it increased even more with age. More importantly, it also promoted differential expression of genes associated with T infiltrated cells between EAE mice of different ages. *IFN-γ*, produced by Th1 cells, *IL-17*, produced by Th17 cells and *IL-10*, produced by Treg cells (Raphael et al. 2015), were found to be upregulated in 12-month-old middle-aged EAE mouse spinal cords. This inflammatory environment seen in middle-aged EAE mice was confirmed by the elevated number of infiltrating CD4 and CD8 T cells in spinal cord lesions, which was more pronounced for the CD8 T cells compared to 3- and 6-month-old EAE mice. Pathologically, MS lesions are characterized by the presence of CD4 and CD8 T cells, as well as macrophages, where CD4 T cells appears to be predominant in acute lesions and CD8 T cells more apparent in chronic lesions (Fletcher et al. 2010; van Langelaar et al. 2020). It is important to mention that CD8 T cells that develop a more cytotoxic immune response are implicated in a more severe MS pathogenesis. In fact, one study showed that EAE induction in young animals by adoptive transfer of CD4^+^ T cells increased the number of infiltrating CD8^+^ T cells in the CNS, exacerbating EAE pathogenesis (Wagner et al. 2019). In the same study, using adoptive transfer of CD8^+^ T cells in the periphery of naive mice, EAE mice developed an atypical EAE course, associated with increased chemokine and cytokine expression (Wagner et al. 2019). These results are in line with our findings leading us to conclude that EAE induction and the atypical EAE course observed in middle-aged EAE mice may be due to an increased number of infiltrating T cells, especially the CD8 subset. An important observation in our study is that, by active immunization of EAE mice, is not only possible to observe that CD4 cells are present in EAE lesions, as previously described (Atkinson et al. 2022), but also that CD8 cells are at a higher level in the middle-aged mice contributing to the cytotoxic effects. Also, although we did not find any differences in distinct animal ages for CD4 and CD8 T cells in both EAE or control groups in the periphery, we did find changes in a specific subset of regulatory T cells – CD4^+^CD25^−^Foxp3^+^. Regulatory T cells play an important role in maintaining tolerance, suppressing excessive inflammation and decreasing T cell migratory capacity to the CNS in MS (Kimura 2020). Interestingly, upon disruption of homeostasis, CD4 T cells recruit a specific subset of regulatory CD25^−^ T cells, which in turn can differentiate and contribute to the pool of regulatory T cells expressing CD25^+^Foxp3^+^ (Zelenay et al. 2005). More recently, another group proposed that the absence of CD25 may account for the diminished suppressive function of CD4^+^CD25^−^Foxp3^+^ cells and hyporesponsiveness to stimulation, classified as exhausted regulatory T cells (Zohouri et al. 2021). Considering these hypotheses, our data suggest that high amounts of these Treg cells can either contribute to the maintenance of an excessive immune response or to the loss of suppressive function and exacerbation of an excessive inflammatory response. Taken together, we hypothesize that the alterations observed in 12-month-old middle-aged EAE mice contributed to the atypical and worse disease outcomes.

It is critical to mention that age is a key factor in the development of worse disease outcomes as middle-aged control mice already exhibit functional differences from the young and adult control group (Ribeiro et al. 2022). This finding might explain the failure of some DMTs in older MS patients and reinforces the potential need to stratify patients by age to apply the most adequate treatment (Macaron et al. 2023). With this in mind, and due to recently published data on the role of pentamidine in the amelioration of S100B toxic effects on EAE mice (Di Sante et al. 2020; Barros et al. 2022), we investigated whether the reduction of neuroinflammation by depletion of S100B in EAE mice at distinct ages could be an effective therapeutic approach for young- or late-onset MS. We found that S100B KO mice presented fewer motor symptoms, especially in 3-month-old EAE mice, accompanied by reduced formation of spinal cord demyelinating lesions. Interestingly, the reduced gliosis upon S100B depletion that has been previously described (Roltsch et al. 2010), was more pronounced in 12-month-old middle-aged S100B KO mice in parallel with upregulation of genes involved in glial neuroprotective activity. Toxic levels of S100B are also known to initiate an inflammatory cascade that worsens pathological features (Langeh and Singh 2020). Targeting S100B could constitute a valuable weapon to control CNS neuroinflammation. Indeed, depletion of S100B in 11-week-old mice resulted in significant attenuation of pro-inflammatory cytokine production upon demyelinating insult in an ex vivo demyelinating model (Barros et al. 2022). Moreover, targeting S100B with pentamidine resulted in increase of microglia with regenerative properties (Barros et al. 2022). Here, we found that S100B depletion reduced levels of *TSPO*, a marker of brain injury and inflammation that has been widely described in the context of MS (Chen et al. 2018; Wood 2021). Moreover, TSPO expression is greatly upregulated in microglia activation (Yao et al. 2020), suggesting that S100B depletion positively contributes to ameliorating microglia activation that was more pronounced in 3- and 6-month-old adult EAE S100B KO mice. S100B depletion also reduced levels of *MHC-II*, an antigen presenting molecule often related to autoimmune diseases and that also triggers inflammatory cascades (Friese et al. 2005; Martin et al. 2021). Similar to TSPO, MHC-II is linked to microglia activation in inflammatory and pathological context of EAE and cuprizone MS models (Wolf et al. 2018). Interestingly, in what concerns phagocytosis genes – *MERTK* and *TREM2* – S100B depletion led to the restoration of microglia phagocytic gene expression in 12-month-old EAE S100B KO when compared to age-matched EAE WT mice with reduction of myelin debris accumulation. This suggests that targeting S100B could be beneficial for improved myelin debris clearance by microglia favoring the remyelination process. Indeed, a previous study from our group described that inhibiting the interaction between S100B and its specific receptor [receptor for advanced glycation end products (RAGE)] had positive effects on inflammation favoring the remyelination processes (Santos et al. 2020). Therefore, microglia phagocytosis capacity in the absence of S100B should be carefully addressed to better understand this. Moreover, the increased NF200 staining observed in 12-month-old EAE S100B KO mice compared to EAE WT counterparts suggests that S100B depletion may also play a role in preserving axonal integrity during aging. Given that NF200 is a key marker of axonal structure and stability (Gafson et al. 2020), its increased expression in S100B KO mice may indicate reduced axonal damage, potentially due to improved microglial function and enhanced myelin debris clearance. This aligns with our findings that S100B depletion restores phagocytic gene expression, suggesting that the absence of S100B creates a more neuroprotective environment, facilitating remyelination and preserving axonal integrity. Since chronic inflammation and inefficient debris clearance are major contributors to remyelination failure in aging (Neumann et al. 2019; Pinto and Fernandes 2020), targeting S100B could represent a promising strategy to mitigate age-related deficits in axonal maintenance and repair. Future studies should further investigate the mechanistic relationship between S100B, neurofilament stability, and microglial-mediated remyelination to explore its therapeutic potential in MS and other demyelinating disorders.

Lastly, immune T cells infiltration showed a marked reduction of CD4 and CD8 T cells in spinal cord lesions of S100B KO mice with a marked impact in 12-month-old mice. Furthermore, associated cytokines revealed some interesting results. In terms of *IL-17* gene expression, we found that 6- and 12-month-old EAE S100B KO mice displayed higher levels of Th17 associated cytokine compared to their respective EAE WT mice. In fact, Th17 cells are deregulated and play a central role in autoimmune disorder pathophysiology, including MS (Murphy et al. 2010). However, *IL-10* gene expression and regulatory T cells at the periphery increased with age in EAE S100B KO mice, which may counterbalance the expression of pro-inflammatory cytokines. Moreover, in 12-month-old EAE S100B KO mice, we found that CD8 T cells decreased when compared to respective WT mice. In accordance, treatment of EAE mice with pentamidine potentiated regulatory mechanisms at the periphery, which favored recovery (Barros et al. 2022). This evidence suggests that the depletion of S100B in 12-month-old mice leads to a more regulatory and less cytotoxic phenotype in these animals, which may suggest a potential strategy to reduce MS-associated pathology in late-onset MS patients.

A caveat of this study is that the experiments only used female mice. In the context of MS, sex differences are well documented with females more likely to develop this disease than males (Coyle 2021). However, late-onset MS patients are more frequently male and tend to have a progressive form of the disease (Capasso et al. 2023). Thus, it will be important to explore the impact of age on MS phenotype and pathology in males as well. Moreover, as we aimed to address pathological features, namely demyelinated lesions, glial reactivity and immune cell infiltration and consequent immune responses, we selected the EAE model to answer our questions. Although it is the most commonly used animal model in MS context, it does not fully reproduce all aspects of MS and other animal models (i.e. viral and toxic models) could be used (Lassmann and Bradl 2017; Boziki et al. 2023). Another key aspect is that few previous studies used EAE mice of different ages, which makes this current study difficult to discuss. As stated previously, one important prior study opened the road to explore this field, however it just addressed the role of a specific type of cells in EAE pathogenesis (Atkinson et al. 2022). Other limitation of our study is the lack of knowledge on the antigen-specific response against MOG in the context of aging and EAE. it is important to note that age-related changes in immune function, particularly in T-cell responses, have been extensively studied in the context of aging and autoimmunity (Cisneros et al. 2022). Research has demonstrated that aging results in an overall dysregulation of the immune system, leading to alterations in both the quantity and functionality of T cells. For example, in aging, there is often an increase in T-cell exhaustion, as well as changes in cytokine profiles and a shift towards more regulatory T-cell responses (Jagger et al. 2014). These age-related changes may impact the antigen-specific response to MOG peptides in different ways, depending on factors such as T-cell repertoire diversity and immune senescence.

In our study, we observed that 12-month-old EAE mice, despite having higher numbers of infiltrating CD4^+^ and CD8^+^ T cells in the CNS, exhibited less severe demyelination compared to the younger groups (3- and 6-month-old). This observation led us to hypothesize that age-related immune alterations, including exhausted T-cell expansion, might play a role in modulating the severity of demyelination, which is consistent with findings from previous studies showing an increase in regulatory T-cell responses with aging (Jagger et al. 2014; Papadopoulos et al. 2020).

While we did not specifically analyze MOG-specific T-cell responses (i.e., through in vitro stimulation with MOG_35–55_ and MOG_37–46_) in this study, existing literature suggests that the MOG-specific response in aging is modulated by immunosenescence (Spatola et al. 2023). Previous studies have shown that aging leads to dysregulated T-cell activation and impaired cytokine production, which may alter the strength and specificity of the T-cell response to MOG peptides. For instance, aging is associated with decreased T-cell activation in response to MOG peptides and a shift towards more regulatory T-cell activity, which could contribute to immunological tolerance rather than the classical autoimmune response seen in younger animals (Bektas et al. 2017; Papadopoulos et al. 2020). In addition, MOG-specific T-cell responses in EAE model have been well characterized in younger animals and have shown that T-cell responses to MOG peptides are critical for initiating autoimmune demyelination (Spatola et al. 2023). However, in the context of aging, these responses are often diminished or altered, making it difficult to draw direct correlations between T-cell infiltration and demyelination severity. This is particularly true given that regulatory mechanisms, such as the upregulation of IL-10 and increased Tregs, are more prominent in older animals and could reduce the efficacy of the MOG-specific immune response. Given the complex interplay between regulatory T-cells, cytokine profiles, and T-cell exhaustion in aging, we believe our current findings provide important insights into the age-related immune modulation in the context of EAE. We also believe that further studies should address this question to understand if in fact aging impacts the antigen responses against MOG.

Due to the increase in life expectancy in society, researchers have started to look more carefully at the effects of age on a disease that used to be associated with young adults. Therefore, more studies should attempt to clarify the underlying mechanisms of MS pathogenesis at different ages. This may justify the inclusion of older patients in clinical trials for new therapies to treat this chronic disorder.

In summary, the results of this study have shown that pathological and molecular alterations differ in mice of different ages in the in vivo active immunization EAE model of MS. Importantly, we described the main alterations when using an S100B KO cohort and how it could be beneficial to translate this into clinical practice. These findings contribute to our understanding of differences in the time of disease onset and its associated worse disease course in older patients. It also highlights the need to find markers that may help clinicians to stratify MS patients and direct them to the best treatment approach to attenuate the pathological hallmarks of their specific disease.

## Supplementary Information

Below is the link to the electronic supplementary material.

Supplementary file1 (DOCX 28414 KB)

## Data Availability

No datasets were generated or analysed during the current study.

## References

[CR1] Absinta M, Maric D, Gharagozloo M, Garton T, Smith MD, Jin J, Fitzgerald KC, Song A, Liu P, Lin J-P, Wu T, Johnson KR, McGavern DB, Schafer DP, Calabresi PA, Reich DS (2021) A lymphocyte–microglia–astrocyte axis in chronic active multiple sclerosis. Nature 597:709–714 34497421 10.1038/s41586-021-03892-7 PMC8719282

[CR2] Acharjee S, Gordon PMK, Lee BH, Read J, Workentine ML, Sharkey KA, Pittman QJ (2021) Characterization of microglial transcriptomes in the brain and spinal cord of mice in early and late experimental autoimmune encephalomyelitis using a RiboTag strategy. Sci Rep 11:14319 34253764 10.1038/s41598-021-93590-1 PMC8275680

[CR3] Aharoni R, Aricha R, Eilam R, From I, Mizrahi K, Arnon R, Souroujon MC, Fuchs S (2013) Age dependent course of EAE in Aire−/− mice. J Neuroimmunol 262:27–34 23849800 10.1016/j.jneuroim.2013.06.001

[CR4] Aharoni R, Eilam R, Arnon R (2021) Astrocytes in Multiple Sclerosis—Essential Constituents with Diverse Multifaceted Functions. Int J Mol Sci 22:5904 34072790 10.3390/ijms22115904 PMC8198285

[CR5] Atkinson JR, Jerome AD, Sas AR, Munie A, Wang C, Ma A, Arnold WD, Segal BM (2022) Biological aging of CNS-resident cells alters the clinical course and immunopathology of autoimmune demyelinating disease. JCI Insight 7:e158153 35511417 10.1172/jci.insight.158153 PMC9309055

[CR6] Ballanti E, Perricone C, Greco E, Ballanti M, Di Muzio G, Chimenti MS, Perricone R (2013) Complement and autoimmunity. Immunol Res 56:477–491 23615835 10.1007/s12026-013-8422-y

[CR7] Barateiro A, Afonso V, Santos G, Cerqueira JJ, Brites D, van Horssen J, Fernandes A (2016) S100B as a Potential Biomarker and Therapeutic Target in Multiple Sclerosis. Mol Neurobiol 53:3976–3991 26184632 10.1007/s12035-015-9336-6

[CR8] Barmpagiannos K, Theotokis P, Petratos S, Pagnin M, Einstein O, Kesidou E, Boziki M, Artemiadis A, Bakirtzis C, Grigoriadis N (2023) The Diversity of Astrocyte Activation during Multiple Sclerosis: Potential Cellular Targets for Novel Disease Modifying Therapeutics. Healthcare 11:1585 37297725 10.3390/healthcare11111585 PMC10253053

[CR9] Barros C, Barateiro A, Neto A, Soromenho B, Basto AP, Mateus JM, Xapelli S, Sebastião AM, Brites D, Graça L, Fernandes A (2022) S100B inhibition protects from chronic experimental autoimmune encephalomyelitis. Brain Commun 4:fcac076 35620168 10.1093/braincomms/fcac076 PMC9128388

[CR10] Bektas A, Schurman SH, Sen R, Ferrucci L (2017) Human T cell immunosenescence and inflammation in aging. J Leukoc Biol 102:977–988 28733462 10.1189/jlb.3RI0716-335R PMC5597513

[CR11] Berard JL, Wolak K, Fournier S, David S (2010) Characterization of relapsing-remitting and chronic forms of experimental autoimmune encephalomyelitis in C57BL/6 mice. Glia 58:434–445 19780195 10.1002/glia.20935

[CR12] Borucki DM, Toutonji A, Couch C, Mallah K, Rohrer B, Tomlinson S (2020) Complement-Mediated Microglial Phagocytosis and Pathological Changes in the Development and Degeneration of the Visual System. Front Immunol 11:566892 33072106 10.3389/fimmu.2020.566892 PMC7541817

[CR13] Boziki M, Theotokis P, Kesidou E, Karafoulidou E, Konstantinou C, Michailidou I, Bahar Y, Altintas A, Grigoriadis N (2023) Sex, aging and immunity in multiple sclerosis and experimental autoimmune encephalomyelitis: An intriguing interaction. Front Neurol 13:1104552 36698908 10.3389/fneur.2022.1104552 PMC9869255

[CR14] Burrows DJ, McGown A, Jain SA, De Felice M, Ramesh TM, Sharrack B, Majid A (2019) Animal models of multiple sclerosis: From rodents to zebrafish. Mult Scler J 25:306–324 10.1177/1352458518805246 30319015

[CR15] Buscarinu MC, Reniè R, Morena E, Romano C, Bellucci G, Marrone A, Bigi R, Salvetti M, Ristori G (2022) Late-Onset MS: Disease Course and Safety-Efficacy of DMTS. Front Neurol 13:829331 35356454 10.3389/fneur.2022.829331 PMC8960027

[CR16] Capasso N, Virgilio E, Covelli A, Giovannini B, Foschi M, Montini F, Nasello M, Nilo A, Prestipino E, Schirò G, Sperandei S, Clerico M, Lanzillo R (2023) Aging in multiple sclerosis: from childhood to old age, etiopathogenesis, and unmet needs: a narrative review. Front Neurol 14:1207617 37332984 10.3389/fneur.2023.1207617 PMC10272733

[CR17] Chavele K-M, Ehrenstein MR (2011) Regulatory T-cells in systemic lupus erythematosus and rheumatoid arthritis. FEBS Lett 585:3603–3610 21827750 10.1016/j.febslet.2011.07.043

[CR18] Chen W-H, Yeh H-L, Tsao C-W, Lien L-M, Chiwaya A, Alizargar J, Bai C-H (2018) Plasma Translocator Protein Levels and Outcomes of Acute Ischemic Stroke: A Pilot Study. Dis Markers 2018:9831079 30034558 10.1155/2018/9831079 PMC6033241

[CR19] Cignarella F, Filipello F, Bollman B, Cantoni C, Locca A, Mikesell R, Manis M, Ibrahim A, Deng L, Benitez BA, Cruchaga C, Licastro D, Mihindukulasuriya K, Harari O, Buckland M, Holtzman DM, Rosenthal A, Schwabe T, Tassi I, Piccio L (2020) TREM2 activation on microglia promotes myelin debris clearance and remyelination in a model of multiple sclerosis. Acta Neuropathol 140:513–534 32772264 10.1007/s00401-020-02193-z PMC7498497

[CR20] Cisneros B, García-Aguirre I, Unzueta J, Arrieta-Cruz I, González-Morales O, Domínguez-Larrieta JM, Tamez-González A, Leyva-Gómez G, Magaña JJ (2022) Immune system modulation in aging: Molecular mechanisms and therapeutic targets. Front Immunol 13:1059173 36591275 10.3389/fimmu.2022.1059173 PMC9797513

[CR21] Clarke LE, Liddelow SA, Chakraborty C, Münch AE, Heiman M, Barres BA (2018) Normal aging induces A1-like astrocyte reactivity. Proc Natl Acad Sci 115:E1896–E1905 29437957 10.1073/pnas.1800165115 PMC5828643

[CR22] Colón Ortiz C, Eroglu C (2024) Astrocyte signaling and interactions in Multiple Sclerosis. Curr Opin Cell Biol 86:102307 38145604 10.1016/j.ceb.2023.102307 PMC10922437

[CR23] Compston A, Coles A (2008) Multiple sclerosis. The Lancet 372:1502–1517 10.1016/S0140-6736(08)61620-7 18970977

[CR24] Constantinescu C, Farooqi N, O’Brien K, Gran B (2011) Experimental autoimmune encephalomyelitis (EAE) as a model for multiple sclerosis (MS). Br J Pharmacol 164:1079–1106 21371012 10.1111/j.1476-5381.2011.01302.x PMC3229753

[CR25] Coyle PK (2021) What Can We Learn from Sex Differences in MS? J Pers Med 11:1006 34683148 10.3390/jpm11101006 PMC8537319

[CR26] Dargahi N, Katsara M, Tselios T, Androutsou M-E, de Courten M, Matsoukas J, Apostolopoulos V (2017) Multiple Sclerosis: Immunopathology and Treatment Update. Brain Sci 7:78 28686222 10.3390/brainsci7070078 PMC5532591

[CR27] Dema M, Eixarch H, Villar LM, Montalban X, Espejo C (2021) Immunosenescence in multiple sclerosis: the identification of new therapeutic targets. Autoimmun Rev 20:102893 34237417 10.1016/j.autrev.2021.102893

[CR28] Dendrou CA, Fugger L, Friese MA (2015) Immunopathology of multiple sclerosis. Nat Rev Immunol 15:545–558 26250739 10.1038/nri3871

[CR29] Di Sante G, Amadio S, Sampaolese B, Clementi ME, Valentini M, Volonté C, Casalbore P, Ria F, Michetti F (2020) The S100B inhibitor pentamidine ameliorates clinical score and neuropathology of relapsing—remitting multiple sclerosis mouse model. Cells 9:748 32197530 10.3390/cells9030748 PMC7140642

[CR30] Distéfano-Gagné F, Bitarafan S, Lacroix S, Gosselin D (2023) Roles and regulation of microglia activity in multiple sclerosis: insights from animal models. Nat Rev Neurosci 24:397–415 37268822 10.1038/s41583-023-00709-6

[CR31] Dobson R, Giovannoni G (2019) Multiple sclerosis – a review. Eur J Neurol 26:27–40 30300457 10.1111/ene.13819

[CR32] Eden D, Gros C, Badji A, Dupont SM, De Leener B, Maranzano J, Zhuoquiong R, Liu Y, Granberg T, Ouellette R, Stawiarz L, Hillert J, Talbott J, Bannier E, Kerbrat A, Edan G, Labauge P, Callot V, Pelletier J, Audoin B, Rasoanandrianina H, Brisset J-C, Valsasina P, Rocca MA, Filippi M, Bakshi R, Tauhid S, Prados F, Yiannakas M, Kearney H, Ciccarelli O, Smith SA, Andrada Treaba C, Mainero C, Lefeuvre J, Reich DS, Nair G, Shepherd TM, Charlson E, Tachibana Y, Hori M, Kamiya K, Chougar L, Narayanan S, Cohen-Adad J (2019) Spatial distribution of multiple sclerosis lesions in the cervical spinal cord. Brain 142:633–646 30715195 10.1093/brain/awy352 PMC6391605

[CR33] Edler MK, Mhatre-Winters I, Richardson JR (2021) Microglia in aging and alzheimer’s disease: A comparative species review. Cells 10:1138 34066847 10.3390/cells10051138 PMC8150617

[CR34] Filippi M, Bar-Or A, Piehl F, Preziosa P, Solari A, Vukusic S, Rocca MA (2018) Multiple sclerosis. Nat Rev Dis Primers 4:43 30410033 10.1038/s41572-018-0041-4

[CR35] Finger CE, Moreno-Gonzalez I, Gutierrez A, Moruno-Manchon JF, McCullough LD (2022) Age-related immune alterations and cerebrovascular inflammation. Mol Psychiatry 27:803–818 34711943 10.1038/s41380-021-01361-1 PMC9046462

[CR36] Fletcher JM, Lalor SJ, Sweeney CM, Tubridy N, Mills KHG (2010) T cells in multiple sclerosis and experimental autoimmune encephalomyelitis. Clin Exp Immunol 162:1–11 20682002 10.1111/j.1365-2249.2010.04143.x PMC2990924

[CR37] Flurkey K, Mcurrer J, Harrison D (2007) Mouse Models in Aging Research. In: The Mouse in Biomedical Research. Elsevier. p 637–672

[CR38] Friese MA, Jones EY, Fugger L (2005) MHC II molecules in inflammatory diseases: interplay of qualities and quantities. Trends Immunol 26:559–561 16139566 10.1016/j.it.2005.08.011

[CR39] Frischer JM, Weigand SD, Guo Y, Kale N, Parisi JE, Pirko I, Mandrekar J, Bramow S, Metz I, Brück W, Lassmann H, Lucchinetti CF (2015) Clinical and pathological insights into the dynamic nature of the white matter multiple sclerosis plaque. Ann Neurol 78:710–721 26239536 10.1002/ana.24497 PMC4623970

[CR40] Gafson A, Barthélemy N, Bomont P, Carare R, Durham H, Julien JP, Kuhle J, Leppert D, Nixon R, Weller RO, Zetterberg H, Matthews P (2020) Neurofilaments: neurobiological foundations for biomarker applications. Brain 143:1975–1998 32408345 10.1093/brain/awaa098 PMC7363489

[CR41] Goverman J (2009) Autoimmune T cell responses in the central nervous system. Nat Rev Immunol 9:393–407 19444307 10.1038/nri2550 PMC2813731

[CR42] Hoogendijk EO, Afilalo J, Ensrud KE, Kowal P, Onder G, Fried LP (2019) Frailty: implications for clinical practice and public health. Lancet 394:1365–1375 31609228 10.1016/S0140-6736(19)31786-6

[CR43] Ingram G, Loveless S, Howell OW, Hakobyan S, Dancey B, Harris CL, Robertson NP, Neal JW, Morgan BP (2014) Complement activation in multiple sclerosis plaques: an immunohistochemical analysis. Acta Neuropathol Commun 2:53 24887075 10.1186/2051-5960-2-53 PMC4048455

[CR44] Jagger A, Shimojima Y, Goronzy JJ, Weyand CM (2014) Regulatory T cells and the immune aging process: A mini-review. Gerontology 60:130–137 24296590 10.1159/000355303 PMC4878402

[CR45] Jahan-Abad AJ, Karima S, Shateri S, Baram SM, Rajaei S, Morteza-Zadeh P, Borhani-Haghighi M, Salari A, Nikzamir A, Gorji A (2020) Serum pro-inflammatory and anti-inflammatory cytokines and the pathogenesis of experimental autoimmune encephalomyelitis. Neuropathology 40:84–92 31709666 10.1111/neup.12612

[CR46] Kaneko S, Wang J, Kaneko M, Yiu G, Hurrell J, Chitnis T, Khoury S, He Z (2006) Protecting axonal degeneration by increasing nicotinamide adenine dinucleotide levels in experimental autoimmune encephalomyelitis models. J Neurosci 26:9794–9804 16988050 10.1523/JNEUROSCI.2116-06.2006 PMC6674451

[CR47] Kimura K (2020) Regulatory T cells in multiple sclerosis. Clin Exp Neuroimmunol 11:148–155

[CR48] Kis B, Rumberg B, Berlit P (2008) Clinical characteristics of patients with late-onset multiple sclerosis. J Neurol 255:697–702 18283394 10.1007/s00415-008-0778-x

[CR49] Kotter MR (2006) Myelin impairs CNS remyelination by inhibiting oligodendrocyte precursor cell differentiation. J Neurosci 26:328–332 16399703 10.1523/JNEUROSCI.2615-05.2006 PMC6674302

[CR50] Langeh U, Singh S (2020) Targeting S100B protein as a surrogate biomarker and its role in various neurological disorders. Curr Neuropharmacol 19:265–277 10.2174/1570159X18666200729100427 PMC8033985 32727332

[CR51] Laser-Azogui A, Kornreich M, Malka-Gibor E, Beck R (2015) Neurofilament assembly and function during neuronal development. Curr Opin Cell Biol 32:92–101 25635910 10.1016/j.ceb.2015.01.003

[CR52] Lassmann H, Bradl M (2017) Multiple sclerosis: experimental models and reality. Acta Neuropathol 133:223–244 27766432 10.1007/s00401-016-1631-4 PMC5250666

[CR53] Li X, Li Y, Jin Y, Zhang Y, Wu J, Xu Z, Huang Y, Cai L, Gao S, Liu T, Zeng F, Wang Y, Wang W, Yuan T-F, Tian H, Shu Y, Guo F, Lu W, Mao Y, Mei X, Rao Y, Peng B (2023) Transcriptional and epigenetic decoding of the microglial aging process. Nat Aging 3:1288–1311 37697166 10.1038/s43587-023-00479-x PMC10570141

[CR54] Liddelow SA, Guttenplan KA, Clarke LE, Bennett FC, Bohlen CJ, Schirmer L, Bennett ML, Münch AE, Chung W-S, Peterson TC, Wilton DK, Frouin A, Napier BA, Panicker N, Kumar M, Buckwalter MS, Rowitch DH, Dawson VL, Dawson TM, Stevens B, Barres BA (2017) Neurotoxic reactive astrocytes are induced by activated microglia. Nature 541:481–487 28099414 10.1038/nature21029 PMC5404890

[CR55] Macaron G, Larochelle C, Arbour N, Galmard M, Girard JM, Prat A, Duquette P (2023) Impact of aging on treatment considerations for multiple sclerosis patients. Front Neurol 14:1197212 37483447 10.3389/fneur.2023.1197212 PMC10361071

[CR56] Mancini V, Di Pietro A, Pasquini L (2023) Microglia depletion as a therapeutic strategy: friend or foe in multiple sclerosis models? Neural Regen Res 18:267–272 35900401 10.4103/1673-5374.346538 PMC9396475

[CR57] Martin R, Sospedra M, Eiermann T, Olsson T (2021) Multiple sclerosis: doubling down on MHC. Trends Genet 37:784–797 34006391 10.1016/j.tig.2021.04.012

[CR58] Matejuk A, Hopke C, Vandenbark AA, Hurn PD, Offner H (2005) Middle-age male mice have increased severity of experimental autoimmune encephalomyelitis and are unresponsive to testosterone therapy. J Immunol 174:2387–2395 15699175 10.4049/jimmunol.174.4.2387

[CR59] Michetti F, D’Ambrosi N, Toesca A, Puglisi MA, Serrano A, Marchese E, Corvino V, Geloso MC (2019) The S100B story: from biomarker to active factor in neural injury. J Neurochem 148:168–187 30144068 10.1111/jnc.14574

[CR60] Murphy ÁC, Lalor SJ, Lynch MA, Mills KHG (2010) Infiltration of Th1 and Th17 cells and activation of microglia in the CNS during the course of experimental autoimmune encephalomyelitis. Brain Behav Immun 24:641–651 20138983 10.1016/j.bbi.2010.01.014

[CR61] Musella A, Gentile A, Rizzo FR, De VF, Fresegna D, Bullitta S, Vanni V, Guadalupi L, Bassi MS, Buttari F, Centonze D, Mandolesi G (2018) Interplay between age and neuroinflammation in multiple sclerosis: Effects on motor and cognitive functions. Front Aging Neurosci 10:1–13 30135651 10.3389/fnagi.2018.00238 PMC6092506

[CR62] Neumann B, Segel M, Chalut K, Franklin R (2019) Remyelination and ageing: Reversing the ravages of time. Mult Scler 25:1835–1841 31687878 10.1177/1352458519884006 PMC7682531

[CR63] Niraula A, Sheridan JF, Godbout JP (2017) Microglia priming with aging and stress. Neuropsychopharmacology 42:318–333 27604565 10.1038/npp.2016.185 PMC5143497

[CR64] Nishioka T, Shimizu J, Iida R, Yamazaki S, Sakaguchi S (2006) CD4+CD25+Foxp3+ T cells and CD4+CD25-Foxp3+ T cells in aged mice. J Immunol 176:6586–6593 16709816 10.4049/jimmunol.176.11.6586

[CR65] O’Gorman WE, Dooms H, Thorne SH, Kuswanto WF, Simonds EF, Krutzik PO, Nolan GP, Abbas AK (2009) The initial phase of an immune response functions to activate regulatory T cells. J Immunol 183:332–339 19542444 10.4049/jimmunol.0900691 PMC2753472

[CR66] Palmer AL, Ousman SS (2018) Astrocytes and aging. Front Aging Neurosci 10:337 30416441 10.3389/fnagi.2018.00337 PMC6212515

[CR67] Papadopoulos D, Magliozzi R, Mitsikostas DD, Gorgoulis VG, Nicholas RS (2020) Aging, cellular senescence, and progressive multiple sclerosis. Front Cell Neurosci 14:178 32694983 10.3389/fncel.2020.00178 PMC7338849

[CR68] Peferoen LAN, Breur M, van de Berg S, Peferoen-Baert R, Boddeke EHWGM, van der Valk P, Pryce G, van Noort JM, Baker D, Amor S (2016) Ageing and recurrent episodes of neuroinflammation promote progressive experimental autoimmune encephalomyelitis in Biozzi ABH mice. Immunology 149:146–156 27388634 10.1111/imm.12644 PMC5011681

[CR69] Petzold A (2002) Markers for different glial cell responses in multiple sclerosis: clinical and pathological correlations. Brain 125:1462–1473 12076997 10.1093/brain/awf165

[CR70] Piccio L, Buonsanti C, Mariani M, Cella M, Gilfillan S, Cross AH, Colonna M, Panina-Bordignon P (2007) Blockade of TREM-2 exacerbates experimental autoimmune encephalomyelitis. Eur J Immunol 37:1290–1301 17407101 10.1002/eji.200636837

[CR71] Pinto MV, Fernandes A (2020) Microglial phagocytosis—rational but challenging therapeutic target in multiple sclerosis. Int J Mol Sci 21:5960 32825077 10.3390/ijms21175960 PMC7504120

[CR72] Pinto MV, Santos FMF, Barros C, Ribeiro AR, Pischel U, Gois PMP, Fernandes A (2021) BASHY dye platform enables the fluorescence bioimaging of myelin debris phagocytosis by microglia during demyelination. Cells 10:3163 34831386 10.3390/cells10113163 PMC8620345

[CR73] Ponath G, Park C, Pitt D (2018) The role of astrocytes in multiple sclerosis. Front Immunol 9:217 29515568 10.3389/fimmu.2018.00217 PMC5826071

[CR74] Portela LVC, Tort ABL, Schaf DV, Ribeiro L, Nora DB, Walz R, Rotta LN, Silva CT, Busnello JV, Kapczinski F, Gonçalves CA, Souza DO (2002) The serum S100B concentration is age dependent. Clin Chem 48:950–952 12029017

[CR75] Raphael I, Nalawade S, Eagar TN, Forsthuber TG (2015) T cell subsets and their signature cytokines in autoimmune and inflammatory diseases. Cytokine 74:5–17 25458968 10.1016/j.cyto.2014.09.011 PMC4416069

[CR76] Ribeiro AR, Barros C, Barateiro A, Howlett SE, Fernandes A (2022) Improved assessment of overall health in variably aged murine models of multiple sclerosis with a novel frailty index tool. J Gerontol: Ser A 77:1–9 10.1093/gerona/glab185 34181005

[CR77] Robinson AP, Harp CT, Noronha A, Miller SD (2014) The experimental autoimmune encephalomyelitis (EAE) model of MS: utility for understanding disease pathophysiology and treatment. Handb Clin Neurol 122:173–189 24507518 10.1016/B978-0-444-52001-2.00008-X PMC3981554

[CR78] Rockwood K, Howlett SE, MacKnight C, Beattie BL, Bergman H, Hébert R, Hogan DB, Wolfson C, McDowell I (2004) Prevalence, attributes, and outcomes of fitness and frailty in community-dwelling older adults: Report from the Canadian study of health and aging. J Gerontol: Ser A 59:1310–1317 10.1093/gerona/59.12.1310 15699531

[CR79] Roltsch E, Holcomb L, Young KA, Marks A, Zimmer DB (2010) PSAPP mice exhibit regionally selective reductions in gliosis and plaque deposition in response to S100B ablation. J Neuroinflammation 7:78 21080947 10.1186/1742-2094-7-78 PMC2996465

[CR80] Salas IH, Burgado J, Allen NJ (2020) Glia: victims or villains of the aging brain? Neurobiol Dis 143:105008 32622920 10.1016/j.nbd.2020.105008

[CR81] Santos G, Barateiro A, Gomes CM, Brites D, Fernandes A (2018) Impaired oligodendrogenesis and myelination by elevated S100B levels during neurodevelopment. Neuropharmacology 129:69–83 29126910 10.1016/j.neuropharm.2017.11.002

[CR82] Santos G, Barateiro A, Brites D, Fernandes A (2020) S100B impairs oligodendrogenesis and myelin repair following demyelination through rage engagement. Front Cell Neurosci 14:279 33100970 10.3389/fncel.2020.00279 PMC7500156

[CR83] Schirmer L, Schafer DP, Bartels T, Rowitch DH, Calabresi PA (2021) Diversity and function of glial cell types in multiple sclerosis. Trends Immunol 42:228–247 33593693 10.1016/j.it.2021.01.005 PMC7914214

[CR84] Seo J-E, Hasan M, Han J-S, Kang M-J, Jung B-H, Kwok S-K, Kim H-Y, Kwon O-S (2015) Experimental autoimmune encephalomyelitis and age-related correlations of NADPH oxidase, MMP-9, and cell adhesion molecules: The increased disease severity and blood–brain barrier permeability in middle-aged mice. J Neuroimmunol 287:43–53 26439961 10.1016/j.jneuroim.2015.08.005

[CR85] Song X, Mitnitski A, Rockwood K (2010) Prevalence and 10-Year Outcomes of Frailty in Older Adults in Relation to Deficit Accumulation. J Am Geriatr Soc 58:681–687 20345864 10.1111/j.1532-5415.2010.02764.x

[CR86] Spatola M, Chuquisana O, Jung W, Lopez JA, Wendel E-M, Ramanathan S, Keller CW, Hahn T, Meinl E, Reindl M, Dale RC, Wiendl H, Lauffenburger DA, Rostásy K, Brilot F, Alter G, Lünemann JD (2023) Humoral signatures of MOG-antibody-associated disease track with age and disease activity. Cell Rep Med 4:100913 36669487 10.1016/j.xcrm.2022.100913 PMC9975090

[CR87] Takahashi K, Rochford CDP, Neumann H (2005) Clearance of apoptotic neurons without inflammation by microglial triggering receptor expressed on myeloid cells-2. J Exp Med 201:647–657 15728241 10.1084/jem.20041611 PMC2213053

[CR88] Tatomir A, Talpos-Caia A, Anselmo F, Kruszewski AM, Boodhoo D, Rus V, Rus H (2017) The complement system as a biomarker of disease activity and response to treatment in multiple sclerosis. Immunol Res 65:1103–1109 29116612 10.1007/s12026-017-8961-8 PMC6563602

[CR89] van Langelaar J, Rijvers L, Smolders J, van Luijn MM (2020) B and T cells driving multiple sclerosis: Identity. Mechanisms and Potential Triggers. Front Immunol 11:760 32457742 10.3389/fimmu.2020.00760 PMC7225320

[CR90] Vaughn CB, Jakimovski D, Kavak KS, Ramanathan M, Benedict RHB, Zivadinov R, Weinstock-Guttman B (2019) Epidemiology and treatment of multiple sclerosis in elderly populations. Nat Rev Neurol 15:329–342 31000816 10.1038/s41582-019-0183-3

[CR91] Voß EV, Škuljec J, Gudi V, Skripuletz T, Pul R, Trebst C, Stangel M (2012) Characterisation of microglia during de- and remyelination: Can they create a repair promoting environment? Neurobiol Dis 45:519–528 21971527 10.1016/j.nbd.2011.09.008

[CR92] Wagner CA, Roqué PJ, Mileur TR, Liggitt D, Goverman JM (2019) Myelin-specific CD8+ T cells exacerbate brain inflammation in CNS autoimmunity. J Clin Investig 130:203–213 10.1172/JCI132531 PMC6934187 31573979

[CR93] Webster SD, Yang AJ, Margol L, Garzon-Rodriguez W, Glabe CG, Tenner AJ (2000) Complement Component C1q Modulates the Phagocytosis of Aβ by Microglia. Exp Neurol 161:127–138 10683279 10.1006/exnr.1999.7260

[CR94] Wilkins A (2017) Cerebellar dysfunction in multiple sclerosis. Front Neurol 8:312 28701995 10.3389/fneur.2017.00312 PMC5487391

[CR95] Wood H (2021) TSPO levels in multiple sclerosis lesions reflect microglial density rather than activation state. Nat Rev Neurol 17:462 10.1038/s41582-021-00533-5 34188236

[CR96] Wolf Y, Shemer A, Levy-Efrati L, Gross M, Kim J-S, Engel A, David E, Chappell-Maor L, Grozovski J, Rotkopf R, Biton I, Eilam-Altstadter R, Jung S (2018) Microglial MHC class II is dispensable for experimental autoimmune encephalomyelitis and cuprizone-induced demyelination. Eur J Immunol 48:1308–1318 29697861 10.1002/eji.201847540

[CR97] Yao R, Pan R, Shang C, Li X, Cheng J, Xu J, Li Y (2020) Translocator protein 18 kDa (TSPO) deficiency inhibits microglial activation and impairs mitochondrial function. Front Pharmacol 11:986 32695005 10.3389/fphar.2020.00986 PMC7339871

[CR98] Yoo H-J, Kwon M-S (2022) Aged microglia in neurodegenerative diseases: Microglia lifespan and culture methods. Front Aging Neurosci 13:766267 35069173 10.3389/fnagi.2021.766267 PMC8766407

[CR99] Zelenay S, Lopes-Carvalho T, Caramalho I, Moraes-Fontes MF, Rebelo M, Demengeot J (2005) Foxp3+ CD25- CD4 T cells constitute a reservoir of committed regulatory cells that regain CD25 expression upon homeostatic expansion. Proc Natl Acad Sci U S A 102:4091–4096 15753306 10.1073/pnas.0408679102 PMC554795

[CR100] Zohouri M, Mehdipour F, Razmkhah M, Faghih Z, Ghaderi A (2021) CD4+CD25-FoxP3+ T cells: a distinct subset or a heterogeneous population? Int Rev Immunol 40:307–316 32705909 10.1080/08830185.2020.1797005

