# Supplementary material for: Experimental autoimmune encephalomyelitis pathogenesis alters along animal age: impact of S100B expression
Source: J Neuroimmune Pharmacol. 2025 Apr 14;20(1):37. doi: 10.1007/s11481-025-10195-5 (PMC11997003; doi:10.1007/s11481-025-10195-5)
Supplement: Supplementary file 1 — Supplementary file1 (DOCX 28414 KB) [file 11481_2025_10195_MOESM1_ESM.docx]

**Supplementary material**

**
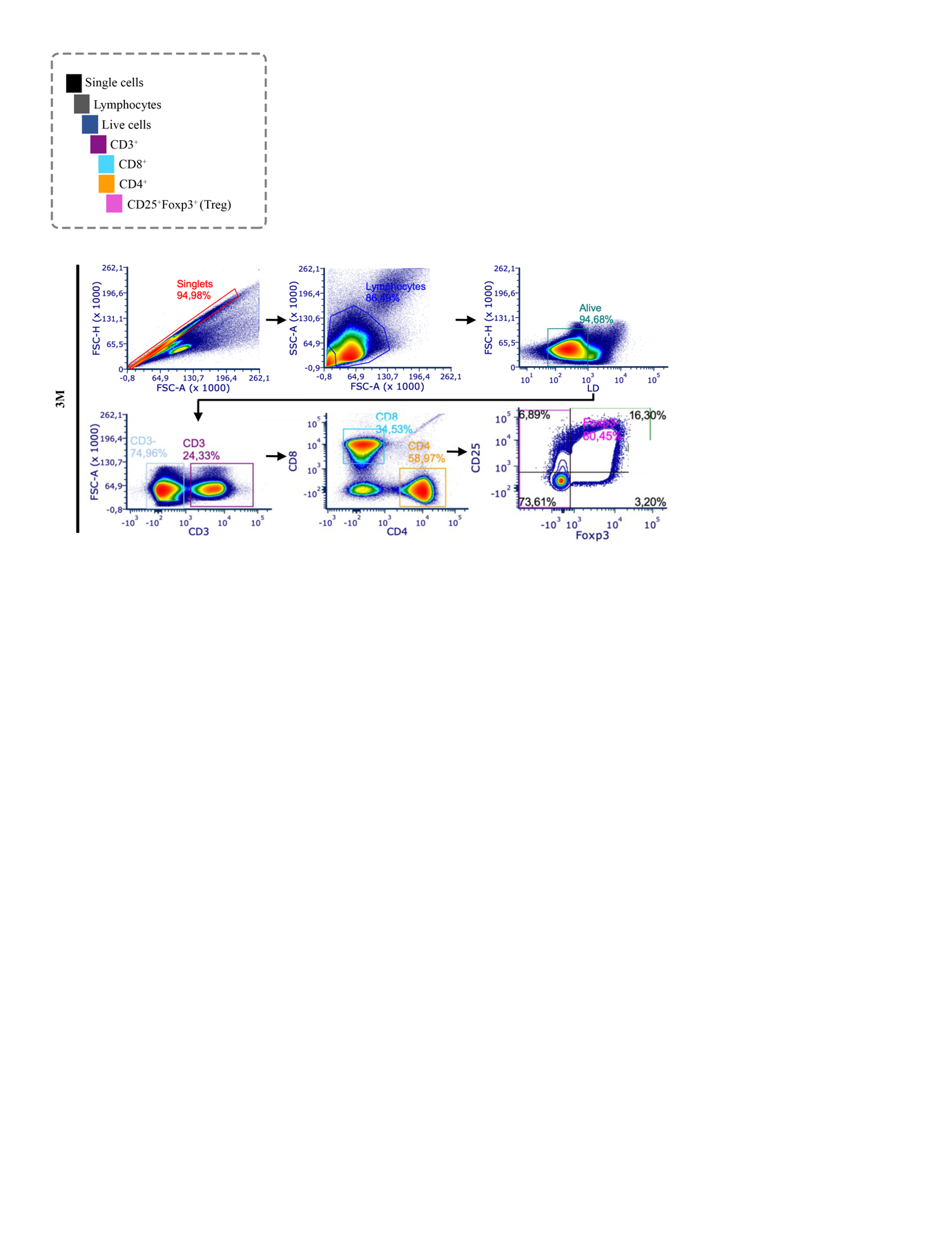
**

**Supplementary Fig. 1: T cell flow cytometry.** Female C57BL/6 mice at 3-, 6- and 12-months-old were induced with EAE by MOG35-55 immunization and followed for 23 days post-EAE induction; spleens were collected at the end of experiment and processed for flow cytometry analysis. T cell gating strategy for CD3, CD8, CD4, and Tregs (CD4^+^ CD25^+^ and Foxp3^+^) cells and representative plots of 3-months-old mice with n = 5 animals per group.

**
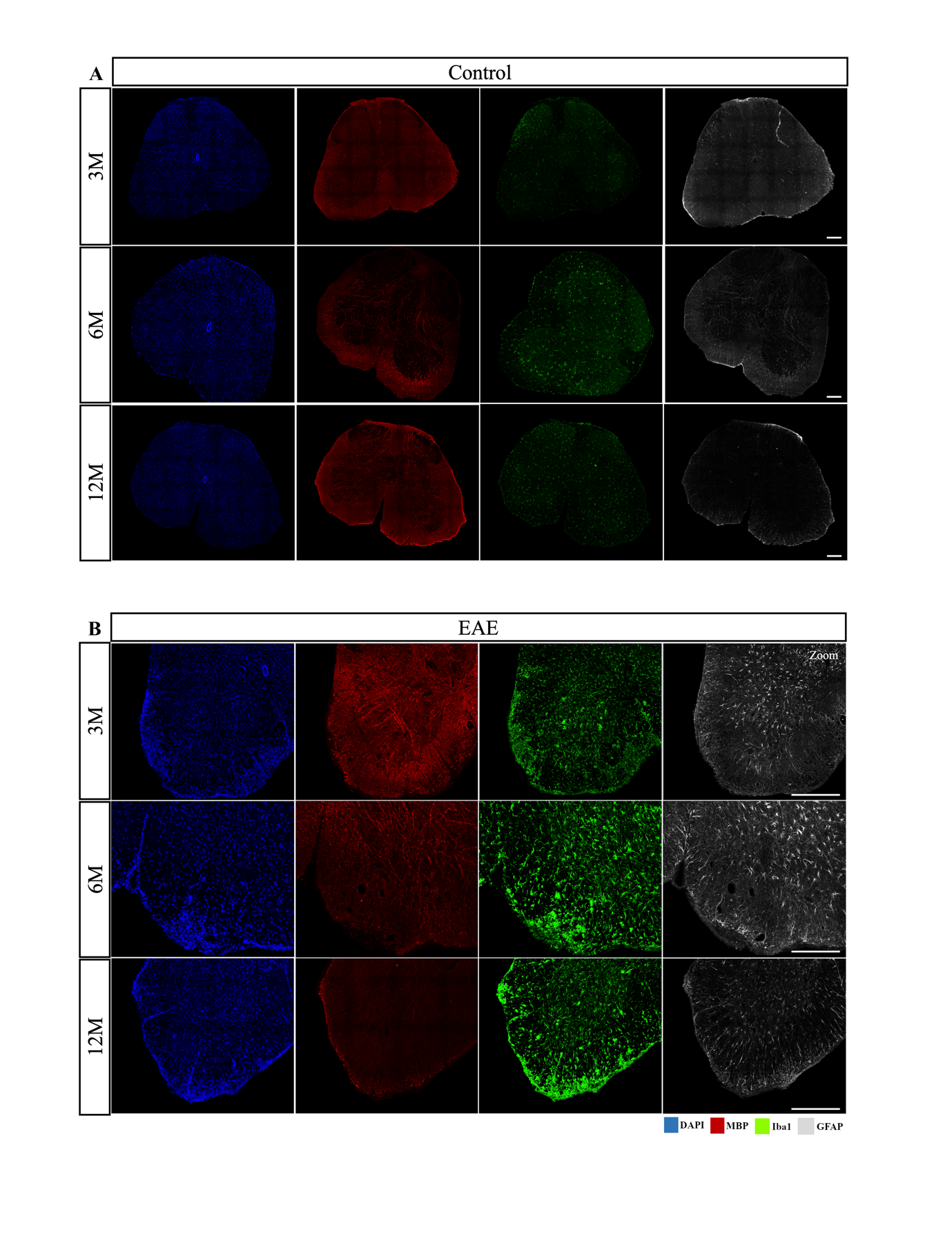
**

**Supplementary Fig. 2: Glial cells staining in demyelinating lesions in the spinal cord.** Female C57BL/6 mice at 3-, 6- and 12-month-old (M) were induced with experimental autoimmune encephalomyelitis (EAE) by MOG_35-55_ immunization and followed for 23 days post-EAE induction; lumbar spinal cords were collected at the end of the experiment and processed for immunohistochemistry. **(A)** Representative images of spinal cord sections of age-matched controls. **(B)** Representative spinal cord zoom sections of demyelinated lesions (without myelin) of EAE mice. Nuclei were identified by DAPI (blue), myelin by MBP (red), microglia by Iba1 (green) and astrocytes by GFAP (white). Scale bar: 100 µm. Magnification: 20x. EAE - Experimental autoimmune encephalomyelitis; MBP – Myelin basic protein; Iba1 - Ionized calcium binding adaptor molecule 1; GFAP - Glial fibrillary acidic protein.

**
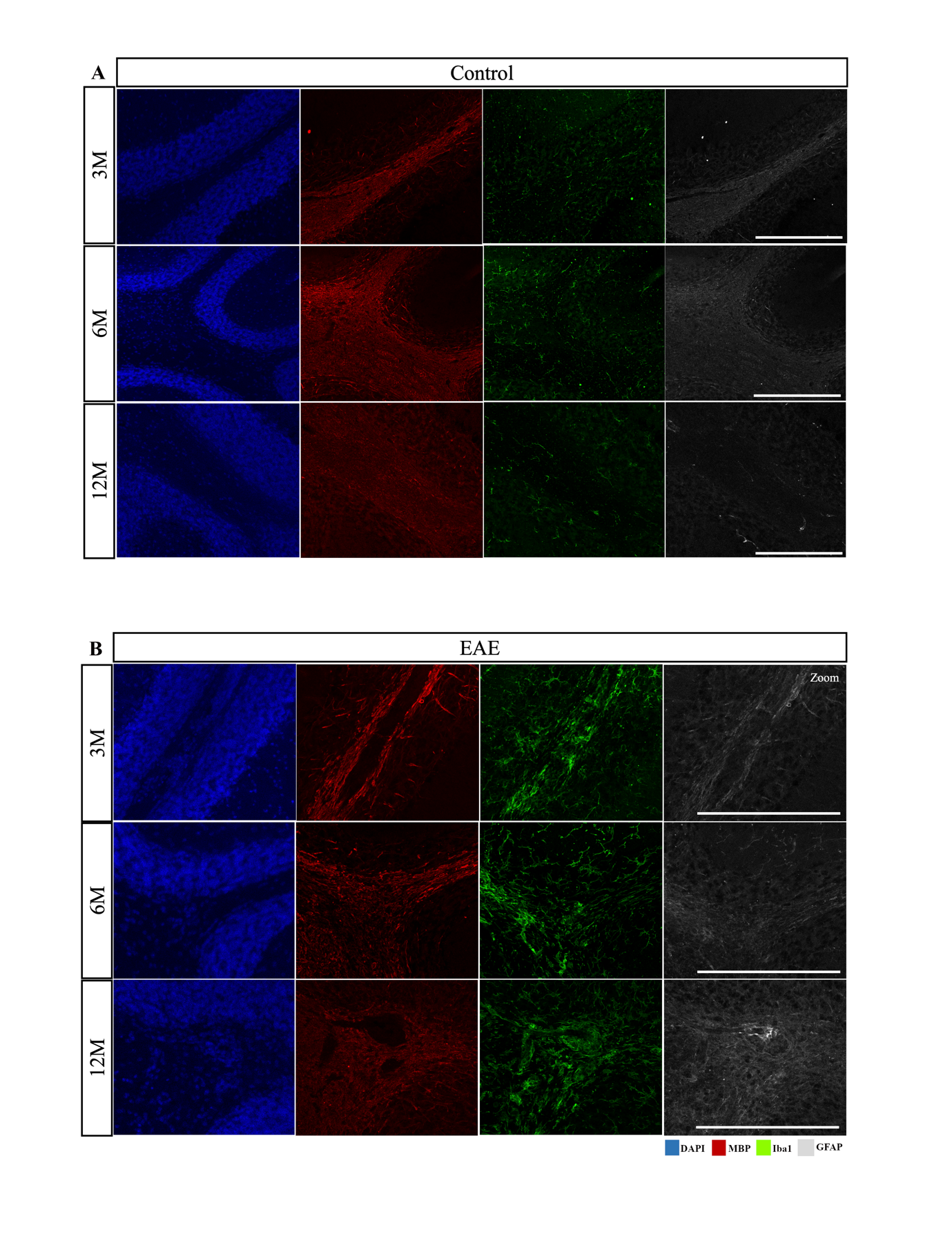
**

**Supplementary Fig. 3: Glial cells staining in demyelinating lesions in the cerebellum.** Female C57BL/6 mice at 3-, 6- and 12-month-old (M) were induced with experimental autoimmune encephalomyelitis (EAE) by MOG_35-55_ immunization and followed for 23 days post-EAE induction; cerebellum was collected at the end of the experiment and processed for immunohistochemistry. **(A)** Representative images of cerebellum sections of age-matched controls. **(B)** Representative cerebellum zoom sections of demyelinated lesions (without myelin) of EAE mice. Nuclei were identified by DAPI (blue), myelin by MBP (red), microglia by Iba1 (green) and astrocytes by GFAP (white). Scale bar: 100 µm. Magnification: 20x. EAE - Experimental autoimmune encephalomyelitis; MBP – Myelin basic protein; Iba1 - Ionized calcium binding adaptor molecule 1; GFAP - Glial fibrillary acidic protein.

**
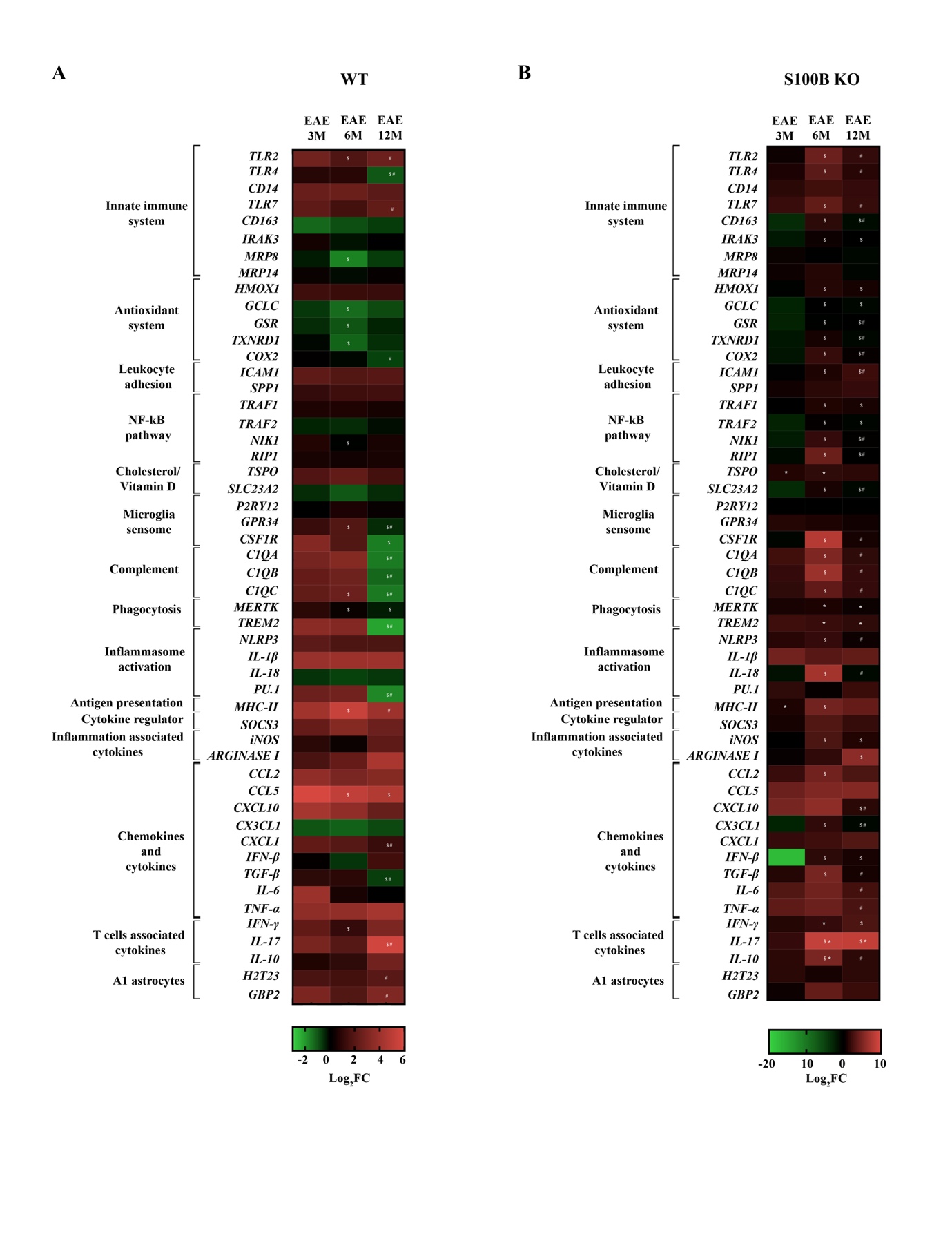
Supplementary Fig. 4: EAE induction induced an age-specific gene expression profile in the spinal cord of WT and S100B KO mice.** Female S100B KO and WT C57BL/6 mice at 3-, 6- and 12-months-old were induced with EAE by MOG_35-55_ immunization and followed for 23 days post-EAE induction; thoracic spinal cords were collected at the end of the experiment and processed for gene expression analysis. Heatmap representation of the spinal cord gene profile of EAE WT mice **(A)** and EAE S100B KO mice **(B)**. Results were normalized to endogenous controls (*RPL19* and *RPL29*), and then fold changes of each experimental group were normalized to control 3-months-old WT mice followed by the normalization of the EAE induced mice to the respective control group at the same age. Results are expressed as mean ± SEM of n=5 per group in each experiment and were analysed by Two-way ANOVA with multiple comparisons. EAE - Experimental autoimmune encephalomyelitis. $ *p*<0.05 vs. 3-month-old EAE mice; # *p*<0.05 vs. 6-month-old EAE mice; * *p<*0.05 vs. aged matched EAE WT mice.

**
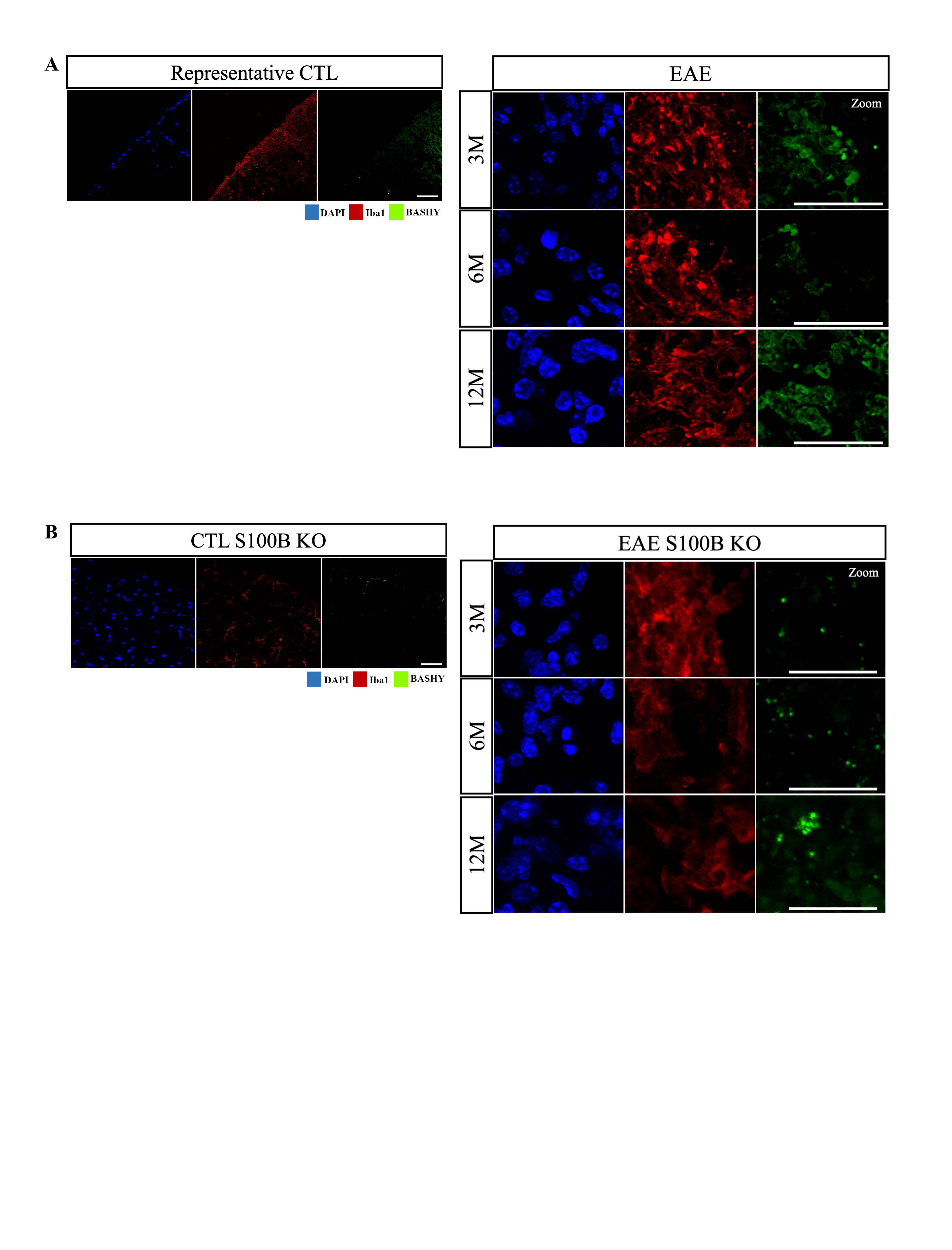
Supplementary Fig. 5: Myelin debris staining and colocalization with microglia in spinal cord lesions.** Female WT C57BL/6 and S100B KO mice at 3-, 6- and 12-month-old (M) were induced with EAE by MOG_35-55_ immunization and followed for 23 days post-EAE induction. Lumbar spinal cords were collected at the end of experiment and processed for immunohistochemistry. **(A)** Representative zoom spinal cord sections of CTL WT and EAE WT mice; and **(B)** CTL S100B KO and EAE S100B KO mice. Nuclei were identified by DAPI (blue), microglia by Iba1 (red) and myelin debris by BASHY probe (green). Scale bar: 100 µm. Magnification: 40x. EAE - Experimental autoimmune encephalomyelitis; CTL – Control; Iba1 - Ionized calcium binding adaptor molecule 1.

**
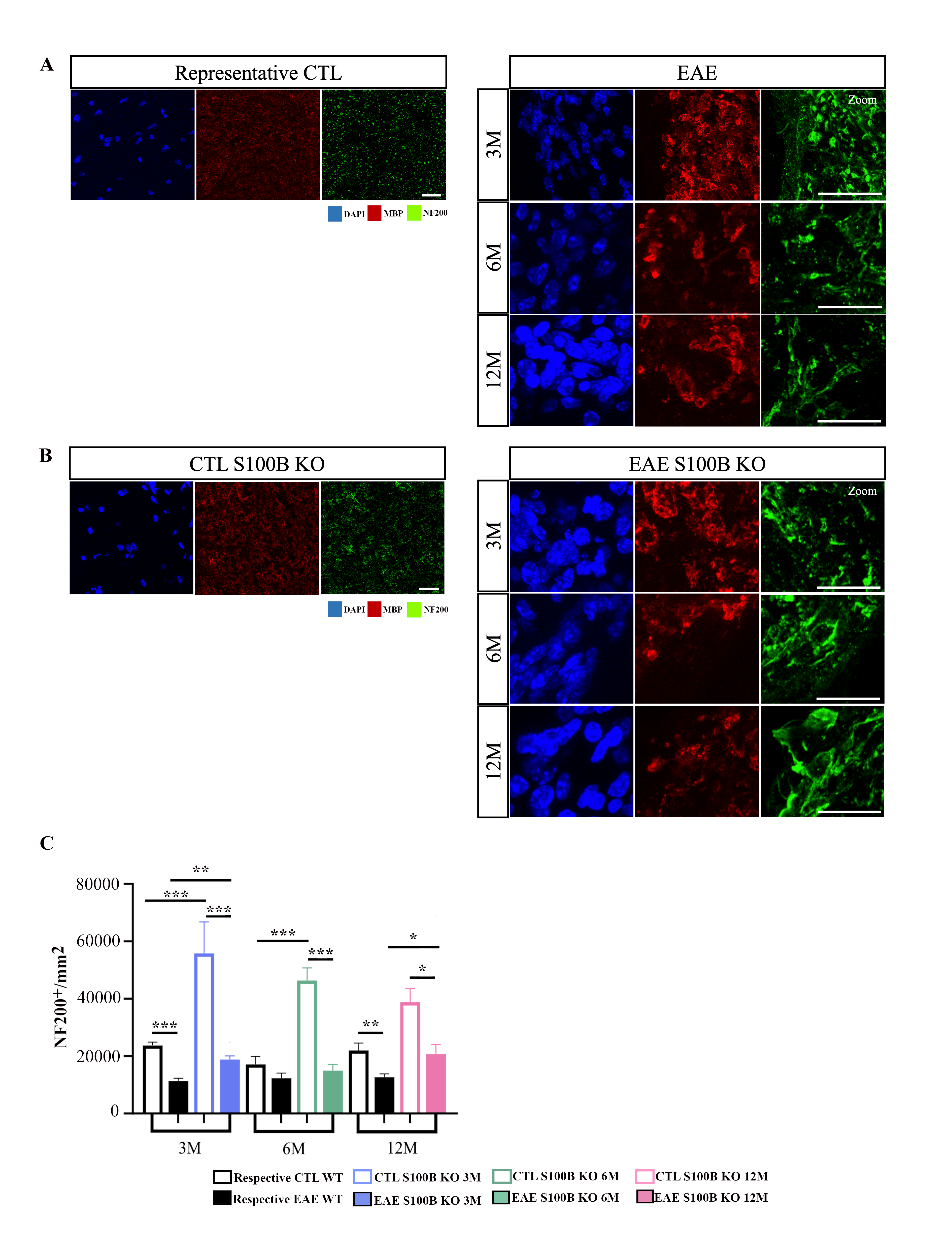
Supplementary Fig. 6: Neurofilaments staining in demyelinating lesions in the spinal cord.** Female WT C57BL/6 and S100B KO mice at 3-, 6- and 12-month-old (M) were induced with EAE by MOG_35-55_ immunization and followed for 23 days post-EAE induction. Lumbar spinal cords were collected at the end of experiment and processed for immunohistochemistry. **(A)** Representative zoom spinal cord sections of demyelinated lesions (without myelin) of CTL WT and EAE WT mice; and **(B)** CTL S100B KO and EAE S100B KO mice. Nuclei were identified by DAPI (blue), myelin by MBP (red) and neurofilaments by NF200 (green). **(C)** Graph bar represents the number of NF200^+^ cells counted (green) in NAWM. Results are expressed as mean ± SEM of one independent experiment, n=4-5 per group for each experiment and were analysed by Two-way ANOVA with multiple comparisons. EAE - Experimental autoimmune encephalomyelitis; CTL – Control; NAWM – Normal appearing white matter; MBP – Myelin basic protein; NF200 – Neurofilament 200. * *p*<0.05; ** *p*<0.01 and *** *p*<0.001.

**
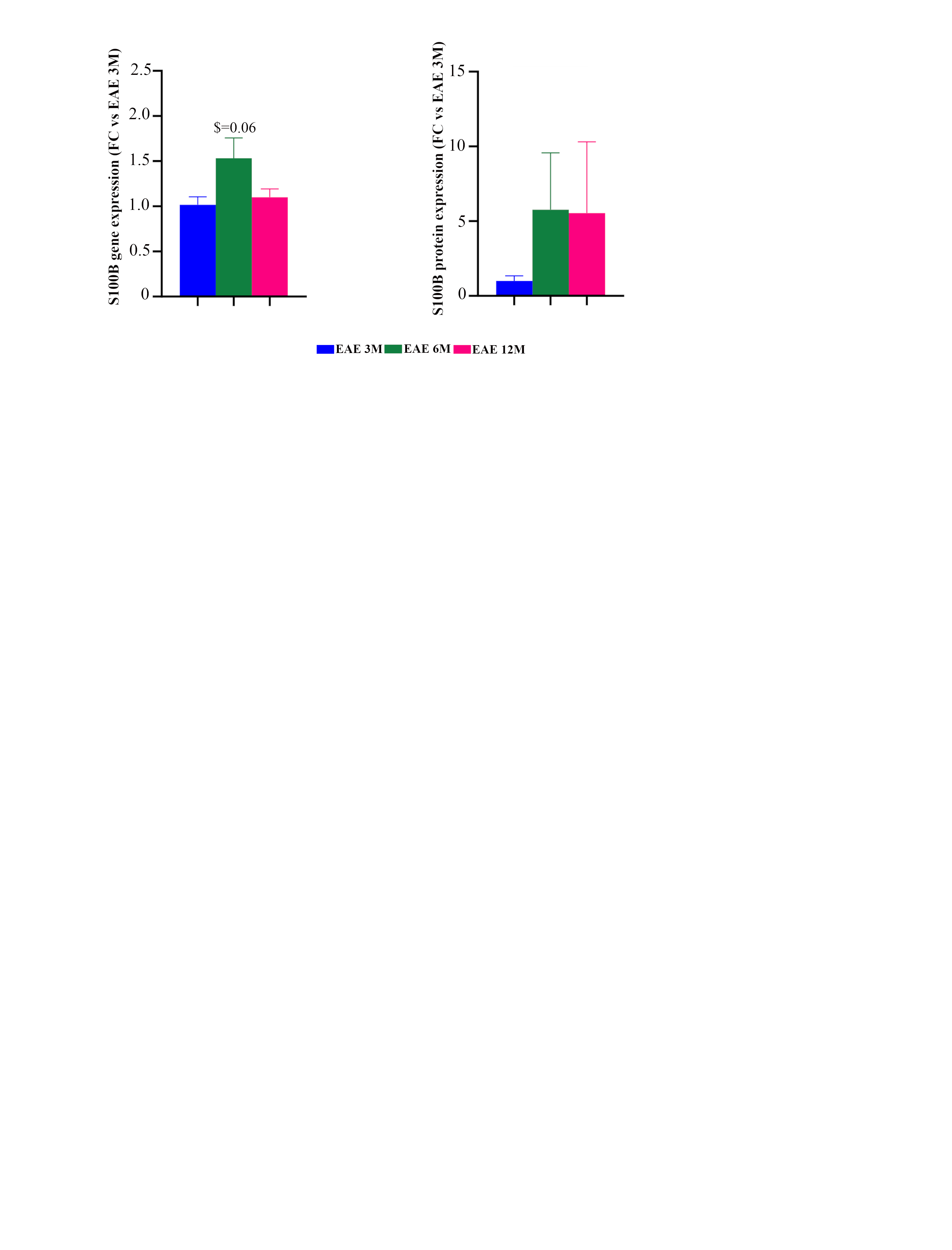
**

**Supplementary Fig. 7: S100B gene and protein expression in the spinal cord of WT EAE mice.** Female C57BL/6 mice at 3-, 6- and 12-month-old (M) were induced with experimental autoimmune encephalomyelitis (EAE) by MOG_35-55_ immunization and followed for 23 days post-EAE induction. Thoracic spinal cords were collected at the end of the experiment and processed for gene and protein expression analysis. Results from gene expression were normalized to endogenous controls (*RPL19*), and then fold changes of each experimental group were normalized to 3-month-old EAE mice. Results from protein expression were normalized to endogenous β-actin and fold changes for each experimental groups were normalized to 3-month-old EAE mice. Results are expressed as mean ± SEM of one independent experiment, n=5 per group for each experiment. Results were analysed by Two-way ANOVA with multiple comparisons. EAE - Experimental autoimmune encephalomyelitis; FC – Fold change. $ *vs.* 3-month-old EAE mice.

**
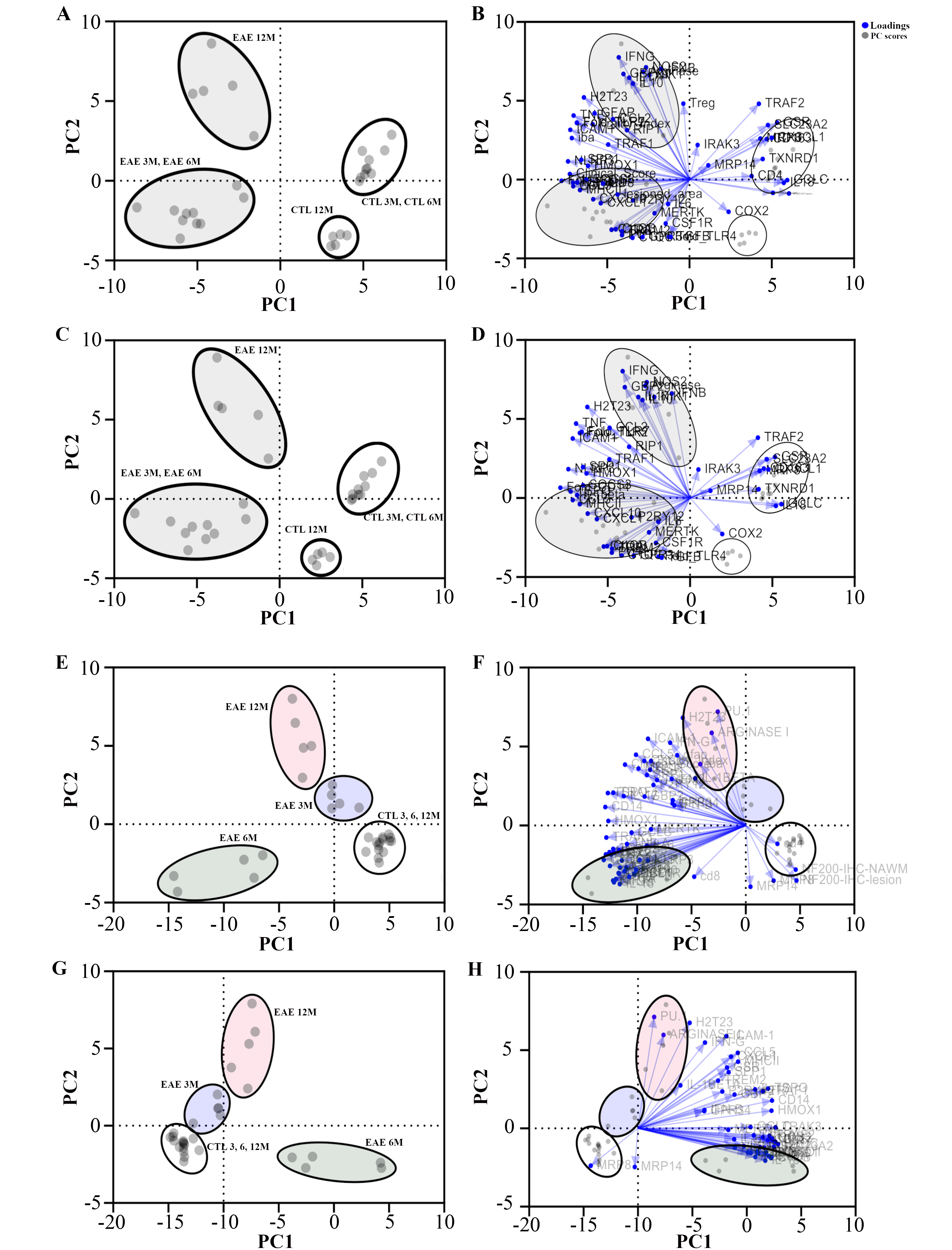
Supplementary Fig. 8: Distinct characteristics linked to mouse age and S100B ablation contribute to a specific age-phenotype in response to EAE induction.** Female S100B KO and WT C57BL/6 mice at 3-, 6- and 12-month-old were induced with EAE by MOG_35-55_ immunization and followed for 23 days post-EAE induction. Graph represents the Principal Component Analysis of the whole set of variables associated with EAE pathogenesis in **(A)** WT and **(E)** S100B KO mice (clinical score, EAE-Clinical Frailty Index, demyelination lesions, glial reactivity, inflammation and peripherical immune response). In **(B)** and **(F)** is represented the loading factors and PC scores of each variable in WT and S100B KO mice respectively. Graph represents the Principal Component Analysis of the gene expression analysis associated with inflammation and glial activity (represented in Supplementary Fig. 4) in **(C)** WT and **(G)** S100B KO mice. In **(D)** and **(H)** is represented the loading factors and PC scores of each gene in WT and S100B KO mice respectively. Colour circles were added manually and correspond to the different mice groups. The specific results of the PCA are represented in Supplementary Table 4 and 5. EAE – Experimental autoimmune encephalomyelitis; CTL – Control; PC1 – Principal component 1; PC2 – Principal component 2.

**Supplementary Table 1: List of genes and respective primer sequences.**

| Gene | NCBI gene ID |  | Sequence (5’-3’) | Amplicon size (bp) |
| --- | --- | --- | --- | --- |
| *TLR2* | 24088 | Sense  Antisense | TGTACCGCAACAGCTTCAGG  TGCTTTCCTGCTGAAGATTT | 197 |
| *TLR4* | 21898 | Sense  Antisense | GTGCCAGAGACATTGCAGAA  ACCTGGCTGGTTTACACGTC | 201 |
| *CD14* | 12475 | Sense  Antisense | CCCGCAGTGAATTGTGACTA  GCTCAACTTTTCCTGCGAAC | 242 |
| *TLR7* | 170743 | Sense  Antisense | TTGCAAAGAAAGCGATTGTG  TGGAAATTTTGGACCTCAGC | 262 |
| *CD163* | 93671 | Sense  Antisense | AGCTCCACTCTTCCCTCACA  TGGTGTGCAGGGAATTACAA | 253 |
| *IRAK3* | 73914 | Sense  Antisense | CCATCAGTTCCATGAGGAGGTC  TGGAGGTTCTAACGGGCTG | 81 |
| *MRP8* | 20201 | Sense  Antisense | CACTAGGCAAGCAAGTGAGGT  GCGCGGACAAATGACTCTG | 63 |
| *MRP14* | 20202 | Sense  Antisense | TCCATAGATGTCATTTATGAGGGC  ATACTCTAGGAAGGAAGGACACC | 130 |
| *HMOX1* | 15368 | Sense  Antisense | CGCTCTATCTCCTCTTCCAGGGC  GAAGGGTCAGGTGTCCAGAGAAGG | 87 |
| *GCLC* | 14629 | Sense  Antisense | TGGCACATTGATGACAACCT  CACAGACCCAACCCAGAG | 141 |
| *GSR* | 14782 | Sense  Antisense | CAGCATAGACGCCTTTGACA  CACGACCATGATTCCAGATG | 161 |
| *TXNRD1* | 50493 | Sense  Antisense | CTCTTCCTACCGCCAGCAACACTG  CAGGGTGACTGCTCAATCCACAAAC | 80 |
| *COX2* | 17709 | Sense  Antisense | CCCCAAAGATAGCATCTGGA  GCTGTACAAGCAGTGGCAAA | 232 |
| *ICAM1* | 15894 | Sense  Antisense | GTCTGCTGAGACCCCTCTTG  CGAAGGTGGTTCTTCTGAGC | 238 |
| *SPP1* | 20750 | Sense  Antisense | GTGAGATTCGTCAGATTCATCCG  AGCAAGAAACTCTTCCAAGCAA | 134 |
| *TRAF1* | 22029 | Sense  Antisense | GCAGTGTAGAAAGCTGGAGAG  AGGGTGGTGGAATTACAGCAA | 194 |
| *TRAF2* | 22030 | Sense  Antisense | GTGCATCCATCATTGGGACAG  AGAGAGTAGTTCGGCCTTTCC | 84 |
| *NIK* | 53859 | Sense  Antisense | GGCTGAACTCTTGGCTATTCTCA  TGTGGGAAGTGGGAGATCCTA | 127 |
| *RIP1* | 19766 | Sense  Antisense | CCAGTAGCTTCACCACTCGAC  GAAGACAGACCTAGACAGCGG | 182 |
| *TSPO* | 12257 | Sense  Antisense | GCCAGGTAAGGGTACAGCAA  TGGGAGGTTTCACAGAGGAC | 214 |
| *SLC23A2* | 54338 | Sense  Antisense | AACACTAGGAAAATCGTCAG  CACTGATAGAAGTGGTCAT | 175 |
| *P2RY12* | 70839 | Sense  Antisense | CAGGACGGTGTACAGCAATG  CACCTCAGCCAATACCACCT | 100 |
| *GPR34* | 23890 | Sense  Antisense | CCGGGCTGTTGTAGCATATT  GGTTGCTCTTGCTGGATTTC | 259 |
| *CSF1R* | 12978 | Sense  Antisense | GATGTCCCTAGCCAGTCCAA  CCCTAGGACAAAGCAAGCAG | 905 |
| *C1qA* | 12259 | Sense  Antisense | ATTCCCCTGGGTCTCCTTTA  GTGTGCTGACCATGACCCTA | 186 |
| *C1qB* | 12260 | Sense  Antisense | GGTCCCCTTTCTCTCCAAAC  AGACACAGTGGGGTGAGGTC | 228 |
| *C1qC* | 12262 | Sense  Antisense | TGTATCGGCCCTCCACAC  GAGGACCCAAGGGTCAGAAG | 138 |
| *MERTK* | 17289 | Sense  Antisense | GGGAGTAGCCATCAAAACCA  GCCCACAATGACAAAGGACT | 148 |
| *TREM2* | 83433 | Sense  Antisense | CCTGGCTGGACTTAAGCTGT  AACTTCAGATCCTCACTGGACC | 80 |
| *NLRP3* | 216799 | Sense  Antisense | ACAAGCCTTTGCTCCAGACCCTAT  TGCTCTTCACTGCTATCAAGCCCT | 85 |
| *IL-1β* | 16176 | Sense  Antisense | GGTGGAGGCTTTCAGCTCATA  CAGGCTCCGAGATGAACAAC | 67 |
| *IL-18* | 16173 | Sense  Antisense | TTCCTGGGCCAAGAGGAAGTG  TGGTTCCATGCTTTCTGGACTCCT | 132 |
| *PU.1* | 20375 | Sense  Antisense | TTTCTTCACCTCGCCTGTCT  CAGTTCTCGTCCAAGCACAA | 132 |
| *MHC-II* | 111364 | Sense  Antisense | GGTCACCCAGCACACCACTT  TGGGCACCATCTTCATCATTC | 131 |
| *SOCS3* | 12702 | Sense  Antisense | ACCTTTGACAAGCGGACTCTC  CCCTTGCAGTTCTAAGTTCAACA | 101 |
| *iNOS* | 18126 | Sense  Antisense | GTGGACGGGTCGATGTCAC  GTTCTCAGCCCAACAATACAAGA | 127 |
| *ARGINASE 1* | 11846 | Sense  Antisense | GGAGAAGGCGTTTGCTTAGTTC  CTTGGCTTGCTTCGGAACTC | 146 |
| *CCL2* | 20296 | Sense  Antisense | TCTGGACCCATTCCTTCTTG  ATCCCAATGAGTAGGCTGGA | 127 |
| *CCL5* | 20304 | Sense  Antisense | CCCACTTCTTCTCTGGGTTG  GTGCCCACGTCAAGGAGTA | 111 |
| *CXCL10* | 15945 | Sense  Antisense | GGCTCGCAGGGATGATTTCAA  CCAAGTGCTGCCGTCATTTTC | 157 |
| *CX3CL1* | 20312 | Sense  Antisense | TTTCTCCTTCGGGTCAGCAC  CTCACGAATCCCAGTGGCTT | 121 |
| *CXCL1* | 14825 | Sense  Antisense | CAGGGTCAAGGCAAGCCTC  CTGGGATTCACCTCAAGAACATC | 117 |
| *IFN-β* | 15977 | Sense  Antisense | GGCAGTGTAACTCTTCTGCAT  CAGCTCCAAGAAAGGACGAAC | 138 |
| *TGF-β* | 21812 | Sense  Antisense | TTCCTGTTGGCTGAGTTGTG  AAATTGCTCGACGCTGTTCT | 269 |
| *IL-6* | 16193 | Sense  Antisense | GGAAATTGGGGTAGGAAGGA  CCGGAGAGGAGACTTCACAG | 421 |
| *TNF-α* | 21926 | Sense  Antisense | CAGCCTTGTCCCTTGAAGAGAACC  TACTGAACTTCGGGGTGATTGGTCC | 247 |
| *IFN-γ* | 15978 | Sense  Antisense | TGGGTTGTTGACCTCAAACTTGGC  GGCCATCAGCAACAACATAAGCGT | 118 |
| *IL-17* | 16171 | Sense  Antisense | GGGTCTTCATTGCGGTGGAGAG  ATCCCTCAAAGCTCAGCGTGTC | 170 |
| *IL-10* | 16153 | Sense  Antisense | GCAGCTCTAGGAGCATGTGG  ATGCTGCCTGCTCTTACTGA | 113 |
| *H2T23* | 15040 | Sense  Antisense | GCACCTCAGGGTGACTTCAT  GGACCGCGAATGACATAGC | 212 |
| *GBP2* | 14469 | Sense  Antisense | GGGAAACCTGGGATGAGATT  GGGGTCACTGTCTGACCACT | 285 |
| *S100B* | 20203 | Sense  Antisense | ATGAGCAACCTCTTCGGGTG  GGGGATGAGATCAACGCTC | 189 |

**Supplementary Table 2: Discrimination of EAE-Clinical FI parameters and respective Mean ± SEM values obtained after 23 days of experiments for the WT mice.**

| EAE-Clinical FI parameter | CTL WT 3M | EAE WT 3M | CTL WT 6M | EAE WT 6M | CTL WT 12M | EAE WT 12M |
| --- | --- | --- | --- | --- | --- | --- |
| Kyphosis | 0.14 ± 0.03 | 0.26 ± 0.04 | 0.18 ± 0.03 | 0.48 ± 0.05 | 0.23 ± 0.03 | 0.36 ± 0.05 |
| Tail stiffening | - | 0.38 ± 0.06 | - | 0.52 ± 0.07 | - | 0.28 ± 0.06 |
| Gait | - | 0.38 ± 0.06 | - | 0.42 ± 0.06 | 0.15 ± 0.03 | 0.5 ± 0.05 |
| Body Condition | - | 0.12 ± 0.04 | - | 0.31 ± 0.05 | - | 0.16 ± 0.05 |
| Distended abdomen | - | 0.15 ± 0.04 | - | 0.06 ± 0.02 | - | 0.13 ± 0.04 |
| Tremor | 0.06 ± 0.02 | 0.34 ± 0.05 | 0.02 ± 0.01 | 0.45 ± 0.05 | 0.23 ± 0.03 | 0.45 ± 0.04 |
| Hindlimbs reflex | - | 0.22 ± 0.05 | - | 0.17 ± 0.05 | - | 0.16 ± 0.05 |
| Menace reflex | 0.13 ± 0.04 | 0.10 ± 0.05 | - | 0.12 ± 0.04 | 0.04 ± 0.02 | 0.11 ± 0.04 |
| Forelimb paralysis | - | 0.09 ± 0.04 | - | 0.04 ± 0.02 | - | 0.07 ± 0.03 |
| Body posture | - | 0.15 ± 0.04 | - | 0.34 ± 0.05 | 0.14 ± 0.03 | 0.21 ± 0.04 |
| Nose down | - | 0.16 ± 0.04 | - | 0.33 ± 0.05 | 0.04 ± 0.02 | 0.3 ± 0.05 |
| Forelimb grip strength | - | 0.19 ± 0.04 | - | 0.21 ± 0.05 | 0.14 ± 0.03 | 0.3 ± 0.05 |
| Grid walk | 0.02 ± 0.01 | 0.38 ± 0.05 | 0.06 ± 0.02 | 0.49 ± 0.06 | 0.07 ± 0.02 | 0.4 ± 0.04 |
| Righting test | - | 0.2 ± 0.05 | 0.06 ± 0.02 | 0.2 ± 0.05 | - | 0.18 ± 0.05 |
| Splayed hindlegs | - | 0.2 ± 0.05 | - | 0.36 ± 0.06 | - | 0.22 ± 0.05 |
| Belly drag | - | 0.26 ± 0.05 | - | 0.36 ± 0.06 | 0.05 ± 0.02 | 0.23 ± 0.05 |
| Alopecia | 0.03 ± 0.02 | 0.45 ± 0.02 | 0.19 ± 0.03 | - | 0.18 ± 0.03 | 0.5 ± 0.03 |
| Dermatitis | 0.18 ± 0.04 | 0.09 ± 0.03 | 0.4 ± 0.05 | 0.07 ± 0.02 | 0.18 ± 0.04 | 0.24 ± 0.04 |
| Loss of whiskers | - | 0.05 ± 0.02 | 0.3 ± 0.04 | - | 0.9 ± 0.03 | 0.83 ± 0.05 |
| Coat condition | 0.21 ± 0.03 | 0.17 ± 0.04 | 0.14 ± 0.03 | 0.23 ± 0.04 | 0.18 ± 0.03 | 0.54 ± 0.04 |
| Head tilt | - | 0.11 ± 0.04 | - | - | - | 0.15 ± 0.04 |
| Hearing loss | 0.05 ± 0.02 | - | - | 0.23 ± 0.05 | 0.23 ± 0.05 | 0.43 ± 0.05 |
| Vision loss | - | 0.06 ± 0.03 | 0.1 ± 0.04 | 0.16 ± 0.03 | 0.26 ± 0.04 | 0.43 ± 0.04 |
| Microphthalmia | - | - | - | - | - | 0.16 ± 0.05 |
| Discharge/swollen/  squinting | - | - | - | 0.03 ± 0.02 | - | 0.04 ± 0.02 |
| Nasal discharge | - | - | - | - |  | - |
| Diarrhoea | - | - | - | 0.02 ± 0.01 | - | 0.02 ± 0.01 |
| Rectal prolapse | - | - | - | - | - | - |
| Vaginal prolapse | - | - | - | - | - | 0.03 ± 0.02 |
| Breathing rate | - | 0.11 ± 0.03 | - | 0.06 ± 0.03 | - | 0.18 ± 0.04 |
| Mouse grimace scale | - | 0.4 ± 0.03 | 0.06 ± 0.02 | 0.3 ± 0.05 | 0.09 ± 0.03 | 0.54 ± 0.03 |
| Polierection | 0.38 ± 0.03 | 0.35 ± 0.08 | 0.48 ± 0.02 | 0.41 ± 0.03 | 0.36 ± 0.03 | 0.6 ± 0.07 |
| Temperature | 0.30 ± 0.05 | 0.51 ± 0.06 | 0.05 ± 0.03 | 0.36 ± 0.05 | 0.04 ± 0.01 | 0.36 ± 0.05 |
| Body weight | 0.31 ± 0.03 | 0.36 ± 0.05 | 0.04 ± 0.01 | 0.51 ± 0.05 | 0.06 ± 0.01 | 0.4 ± 0.06 |

(-) Represent values that did not altered along EAE course. Results are presented as mean ± SEM.

**Supplementary Table 3: Discrimination of EAE-Clinical FI parameters and respective Mean ± SEM values obtained after 23 days of experiments for the KO mice.**

| EAE-Clinical FI parameter | CTL KO 3M | EAE KO 3M | CTL KO 6M | EAE KO 6M | CTL KO 12M | EAE KO 12M |
| --- | --- | --- | --- | --- | --- | --- |
| Kyphosis | 0.07 ± 0.02 | 0.18 ± 0.04 | 0.3 ± 0.03 | 0.5 ± 0.05 | 0.41 ± 0.03 | 0.55 ± 0.03 |
| Tail stiffening | - | 0.39 ± 0.07 | - | 0.52 ± 0.07 | - | 0.36 ± 0.06 |
| Gait | 0.37 ± 0.03 | 0.34 ± 0.04 | 0.3 ± 0.03 | 0.6 ± 0.05 | 0.43 ± 0.03 | 0.61 ± 0.03 |
| Body Condition | - | 0.14 ± 0.04 | 0.03 ± 0.01 | 0.29 ± 0.05 | 0.24 ± 0.05 | **0.3 ± 0.04^*^** |
| Distended abdomen | - | **-^*^** | - | **-** | 0.17 ± 0.05 | **0.03 ± 0.02^*^** |
| Tremor | 0.01 ± 0.03 | 0.33 ± 0.05 | 0.18 ± 0.03 | 0.45 ± 0.06 | 0.37 ± 0.03 | 0.6 ± 0.04 |
| Hindlimbs reflex | - | **0.03 ± 0.02^**^** | - | **0.04 ± 0.06^*^** | - | 0.18 ± 0.05 |
| Menace reflex | - | 0.09 ± 0.03 | 0.12 ± 0.03 | 0.23 ± 0.06 | 0.2 ± 0.04 | 0.3 ± 0.04 |
| Forelimb paralysis | - | **-** | - | **-** | - | **-** |
| Body posture | 0.37 ± 0.03 | 0.35 ± 0.04 | 0.47 ± 0.02 | 0.6 ± 0.05 | 0.52 ± 0.01 | 0.61 ± 0.03 |
| Nose down | - | 0.15 ± 0.04 | - | 0.32 ± 0.05 | 0.06 ± 0.03 | 0.32 ± 0.05 |
| Forelimb grip strength | - | 0.31 ± 0.04 | 0.03 ± 0.02 | 0.27 ± 0.05 | 0.24 ± 0.03 | 0.36 ± 0.04 |
| Grid walk | - | **0.26 ± 0.05^*^** | - | 0.44 ± 0.06 | 0.3 ± 0.04 | 0.51 ± 0.05 |
| Righting test | - | 0.02 ± 0.02 | - | 0.07 ± 0.03 | - | 0.14 ± 0.03 |
| Splayed hindlegs | 0.07 ± 0.02 | 0.11 ± 0.04 | - | 0.42 ± 0.06 | 0.12 ± 0.03 | 0.27 ± 0.05 |
| Belly drag | - | **0.11 ± 0.04^*^** | - | 0.3 ± 0.054 | 0.11 ± 0.03 | 0.28 ± 0.05 |
| Alopecia | 0.03 ± 0.02 | 0.03 ± 0.02 | 0.3 ± 0.05 | 0.13 ± 0.03 | - | 0.1 ± 0.03 |
| Dermatitis | 0.13 ± 0.03 | 0.09 ± 0.03 | 0.3 ± 0.05 | 0.14 ± 0.03 | 0.19 ± 0.03 | 0.24 ± 0.04 |
| Loss of whiskers | - | - | - | - | - | - |
| Coat condition | 0.31 ± 0.03 | 0.24 ± 0.04 | 0.37 ± 0.03 | 0.52 ± 0.04 | 0.43 ± 0.03 | 0.51 ± 0.01 |
| Head tilt | - | **-^*^** | - | - | - | **-^*^** |
| Hearing loss | - | 0.03 ± 0.02 | 0.26 ± 0.04 | 0.12 ± 0.04 | 0.41 ± 0.05 | 0.5 ± 0.04 |
| Vision loss | 0.04 ± 0.02 | 0.21 ± 0.04 | 0.4 ± 0.03 | 0.34 ± 0.03 | 0.53 ± 0.02 | 0.62 ± 0.02 |
| Microphthalmia | - | 0.02 ± 0.02 | 0.1 ± 0.03 | 0.02 ± 0.01 | 0.03 ± 0.02 | 0.06 ± 0.03 |
| Discharge/swollen/  squinting | - | - | - | - | 0.02 ± 0.01 | 0.05 ± 0.02 |
| Nasal discharge | - | - | - | - | 0.02 ± 0.01 | - |
| Diarrhoea | - | - | - | - | - | - |
| Rectal prolapse | - | - | - | - | 0.05 ± 0.02 | 0.02 ± 0.01 |
| Vaginal prolapse | - | - | - | - | - | - |
| Breathing rate | - | **0.02 ± 0.02^*^** | - | 0.04 ± 0.02 | - | 0.13 ± 0.03 |
| Mouse grimace scale | - | 0.03 ± 0.02 | 0.05 ± 0.02 | 0.3 ± 0.05 | 0.03 ± 0.02 | **0.23 ± 0.03^*^** |
| Polierection | 0.45 ± 0.02 | 0.33 ± 0.04 | 0.46 ± 0.02 | 0.61 ± 0.04 | 0.49 ± 0.02 | 0.6 ± 0.03 |
| Temperature | 0.23 ± 0.02 | 0.34 ± 0.05 | 0.25 ± 0.04 | 0.45 ± 0.06 | 0.25 ± 0.04 | 0.72 ± 0.05 |
| Body weight | 0.22 ± 0.04 | 0.45 ± 0.05 | 0.43 ± 0.05 | 0.17 ± 0.03 | 0.05 ± 0.01 | - 1. ± 0.04 |

(-) Represent values that did not altered along EAE course. Results are presented as mean ± SEM. Bold values correspond to parameters that are significantly different when compared to respective WT group. * *p*<0.05; ** *p*<0.01.

**Supplementary Table 4: Results of the Principal Component Analysis (PCA) in WT mice.**

| Parameters | PC1 | PC2 |
| --- | --- | --- |
| Clinical_Score | -0.899755307349226 | 0.0296397715682891 |
| Frailty_Index | -0.728283566649078 | 0.297242746842788 |
| TLR2 | -0.84644598361119 | 0.303568327905886 |
| TLR4 | -0.157064518942654 | -0.826982128021019 |
| CD14 | -0.96175607653909 | -0.0183934078457024 |
| TLR7 | -0.846462668951285 | 0.305898501406962 |
| CD163 | 0.704864220403863 | 0.217919666658207 |
| TRAK3 | -0.913297016828768 | 0.0975407513302476 |
| MRP8 | -0.845016733479067 | -0.0432992065637031 |
| MRP14 | 0.860080754754109 | -0.0473888089802394 |
| NLRP3 | -0.896335876663367 | 0.267003277456428 |
| IL-1BETA | -0.823581289578285 | -0.151049500639245 |
| IL-18 | 0.0737725570735855 | 0.1854877827673 |
| PU.1 | 0.646070269597009 | 0.220088348709019 |
| HMOX1 | 0.170982861418189 | 0.0761895519153649 |
| GCLC | -0.327207775343211 | 0.602573311952983 |
| GSR | -0.348259710236941 | 0.578747071057214 |
| TXNRD1 | -0.611678575903216 | 0.188054844726006 |
| COX2 | 0.632529199130613 | 0.40689612521526 |
| ICAM-1 | -0.470450696596557 | 0.265567571480357 |
| SPP1 | -0.302735314950366 | 0.560779330068747 |
| TRAF1 | -0.763170327626644 | 0.0732331692543253 |
| TRAF2 | 0.891680239366757 | -0.00871932219079122 |
| NIK1 | 0.667717955334764 | 0.110240093125635 |
| RIP1 | 0.799135264764068 | 0.30532066007681 |
| TSPO | -0.875836056024433 | -0.0440397126063757 |
| SLC23A2 | 0.356232160984044 | -0.464401316611185 |
| P2RY12 | -0.776497788244877 | -0.00427124394031081 |
| GPR34 | -0.813657612537681 | 0.105838861288006 |
| CSF1R | 0.715222281842549 | 0.292386011270615 |
| C1QA | -0.425199652462359 | -0.299275909764432 |
| C1QB | -0.507571803381882 | -0.800657931265249 |
| C1QC | -0.353262873096259 | -0.830065830676454 |
| MERTK | -0.179270438446227 | -0.634029881080815 |
| TREM2 | -0.586020576625995 | -0.72370892488052 |
| MHCII | -0.554214942561236 | -0.716936018107657 |
| SOCS3 | -0.428759726269158 | -0.839591677095882 |
| iNOS | -0.141847835892267 | -0.827513274666548 |
| ARGINASE I | -0.263053505260711 | -0.485256135675898 |
| CCL2 | -0.509929461103492 | -0.745439580109079 |
| CCL5 | -0.575364131356555 | 0.324070971511292 |
| CXCL10 | -0.873890723789104 | -0.096467003296476 |
| CX3CL1 | -0.213882258028126 | 0.59450476728345 |
| CXCL1 | 0.749538085873049 | 0.224200526382249 |
| IFN-G | -0.723881562422848 | -0.284258262367587 |
| IFN-B | -0.667475912189995 | -0.341049810255018 |
| TGF-B | -0.531920935161658 | 0.656078430454274 |
| IL-6 | -0.453004561444489 | 0.546841178923312 |
| IL-17 | -0.425816783447329 | 0.515687175661837 |
| TNF-A | -0.160857732247575 | -0.353471140584233 |
| IL-10 | -0.793769789914921 | 0.440963623775094 |
| H2T23 | -0.496706875872872 | 0.566880912528086 |
| GBP2 | -0.871501267626095 | 0.343575900417111 |
| Lesioned_area | -0.540592018913604 | -0.215740264091778 |
| Iba1 | -0.88476577778947 | 0.222762282561459 |
| GFAP | -0.713728637807983 | 0.356061312371208 |
| CD4 | 0.566037564219399 | 0.0182824811931525 |
| CD8 | -0.650361087518613 | -0.058254093356703 |
| Treg | -0.046653361202769 | 0.407651422611549 |
| NF200-IHC-lesion | 0.906896074729608 | -0.20115183971223 |
| NF200-IHC-NAWM | 0.760728997824258 | -0.192180635820251 |

**Supplementary Table 5: Results of the Principal Component Analysis (PCA) in S100B KO mice.**

| Parameters | PC1 | PC2 |
| --- | --- | --- |
| Clinical_Score | -0.799935435076944 | 0.454929801325407 |
| Frailty_Index | -0.627723487170231 | 0.482227328762449 |
| TLR2 | -0.832631368950602 | -0.33302230726127 |
| TLR4 | -0.82098719657046 | -0.276019985905112 |
| CD14 | -0.930503693541315 | 0.137657356820433 |
| TLR7 | -0.926979515578795 | -0.228406077722143 |
| CD163 | -0.791757729923177 | -0.330533167754297 |
| TRAK3 | -0.923564738233313 | -0.095108406606471 |
| MRP8 | 0.396046261751797 | -0.435322905960817 |
| MRP14 | 0.066780219839128 | -0.483559770665875 |
| NLRP3 | -0.649451540490049 | -0.277757272401863 |
| IL-1BETA | -0.334819190475284 | 0.351398423783393 |
| IL-18 | -0.83466059835088 | -0.462670732169091 |
| PU.1 | -0.185351462406565 | 0.850072453645417 |
| HMOX1 | -0.910240125279564 | 0.0336354827821731 |
| GCLC | -0.756576255300224 | -0.0563058078200075 |
| GSR | -0.633440311115104 | 0.416289158828056 |
| TXNRD1 | -0.842449201120167 | -0.375417092777394 |
| COX2 | -0.89193856584516 | -0.228995894113611 |
| ICAM-1 | -0.647688123549389 | 0.647668762181022 |
| SPP1 | -0.654493913379938 | 0.377289826322359 |
| TRAF1 | -0.877786604083324 | 0.245168281881444 |
| TRAF2 | -0.878322572588679 | -0.185359362347383 |
| NIK1 | -0.903001880310444 | -0.211080934946104 |
| RIP1 | -0.878876936015871 | -0.434764093349468 |
| TSPO | -0.913668096292557 | 0.242357682249122 |
| SLC23A2 | -0.899394862669294 | -0.31931351511068 |
| P2RY12 | -0.611612361976997 | 0.30238466387329 |
| GPR34 | -0.485638698340198 | 0.164768457864919 |
| CSF1R | -0.739173059730989 | -0.382837084934281 |
| C1QA | -0.85976144510415 | -0.423093594233741 |
| C1QB | -0.872926124182221 | -0.448353763534609 |
| C1QC | -0.940538128267943 | -0.271715712066689 |
| MERTK | -0.627839685134006 | -0.0298587660849791 |
| TREM2 | -0.588229850967514 | 0.3400058606757 |
| MHCII | -0.710398443791637 | 0.42507405538183 |
| SOCS3 | -0.838652377271913 | -0.195621801570997 |
| iNOS | -0.70324771034918 | -0.272185100252252 |
| ARGINASE I | -0.225654993152266 | 0.692126264223493 |
| CCL2 | -0.873074017226273 | -0.213868220168352 |
| CCL5 | -0.727233088536787 | 0.528559483307227 |
| CXCL10 | -0.80267359838223 | -0.360701103335516 |
| CX3CL1 | -0.840823933133849 | -0.379892190407172 |
| CXCL1 | -0.67203042120352 | 0.482391602519034 |
| IFN-G | -0.500680464570684 | 0.618712852739317 |
| IFN-B | -0.473446743788469 | 0.150645318582813 |
| TGF-B | -0.772309808817563 | -0.40511736919211 |
| IL-6 | -0.655221100213575 | -0.161241358738188 |
| IL-17 | -0.808174515867435 | 0.218924988767221 |
| TNF-A | -0.786739358982043 | -0.146474548813578 |
| IL-10 | -0.72701486744157 | -0.349127303013448 |
| H2T23 | -0.417364043502904 | 0.804705283404799 |
| GBP2 | -0.670327793528746 | 0.216291573921232 |
| Lesioned_area | -0.48374263009973 | 0.188632989043624 |
| Iba1 | -0.301277089508261 | 0.4581023110016 |
| GFAP | -0.454339559500882 | 0.525123406326208 |
| CD4 | 0.45041051098945 | -0.144472999687667 |
| CD8 | -0.341559999411612 | -0.404467885597933 |
| Treg | -0.490586459978066 | 0.347981463608503 |
| NF200-IHC-lesion | 0.721437262526017 | -0.434252147266952 |
| NF200-IHC-NAWM | 0.710045250206672 | -0.348395597445516 |
